# Effects of chronic fluoxetine treatment on anxiety- and depressive-like behaviors in adolescent rodents – systematic review and meta-analysis

**DOI:** 10.1007/s43440-022-00420-w

**Published:** 2022-09-24

**Authors:** Joanna Kryst, Iwona Majcher-Maślanka, Agnieszka Chocyk

**Affiliations:** 1 Faculty of Physiotherapy, Institute for Basics Sciences, University of Physical Education, Jana Pawła II Av. 78, 31-571 Kraków, Poland; 2 Department of Pharmacology, Laboratory of Pharmacology and Brain Biostructure, Maj Institute of Pharmacology, Polish Academy of Sciences, Smętna Street 12, 31-343 Kraków, Poland

**Keywords:** Adolescence, Fluoxetine, Anxiety, Depression, Meta-analysis, Rodents

## Abstract

**Background:**

Drugs prescribed for psychiatric disorders in adolescence should be studied very extensively since they can affect developing and thus highly plastic brain differently than they affect the adult brain. Therefore, we aimed to summarize animal studies reporting the behavioral consequences of chronic exposure to the most widely prescribed antidepressant drug among adolescents i.e., fluoxetine.

**Methods:**

Electronic databases (Medline via Pubmed, Web of Science Core Collection, ScienceDirect) were systematically searched until April 12, 2022, for published, peer-reviewed, controlled trials concerning the effects of chronic fluoxetine administration vs. vehicle on anxiety and depression measures in naïve and stress-exposed adolescent rodents. All of the relevant studies were selected and critically appraised, and a meta-analysis of eligible studies was performed.

**Results:**

A total of 18 studies were included in the meta-analysis. In naïve animals, chronic adolescent fluoxetine administration showed dose-related anxiogenic-like effects, measured as a reduction in time spent in the open arms of the elevated plus maze. No significant effects of chronic adolescent fluoxetine on depression-like behavior were reported in naïve animals, while in stress-exposed rodents chronic adolescent fluoxetine significantly decreased immobility time in the forced swim test compared to vehicle.

**Conclusions:**

These results suggest that although chronic fluoxetine treatment proves positive effects in animal models of depression, it may simultaneously increase anxiety in adolescent animals in a dose-related manner. Although the clinical implications of the data should be interpreted with extreme caution, adolescent patients under fluoxetine treatment should be closely monitored.

**Supplementary Information:**

The online version contains supplementary material available at 10.1007/s43440-022-00420-w.

## Introduction

Major depressive disorder (MDD), which is one of the most common psychiatric syndromes among adults, also affects younger populations, and the number of youth prescribed antidepressants has increased [1]. To clarify the following depression terminology, it should be noted that MDD excludes bipolar depression while major depressive episode (MDE) includes also a depressive episode being a part of bipolar disorder [2]. In 2020, 17% of U.S. adolescents aged 12 to 17 years old had at least one MDE, while 41,6% of them received pharmacological treatment [3]. It is a significant increase in the prevalence of depression symptoms among adolescents compared to the year 2017 when MDE was reported among 13,3% of youth aged 12 to 17 years old [4].

Comorbidity in depression is often reported, most often as co-occurrence with anxiety disorders, such as panic disorder, social phobia or generalized anxiety disorder [5]. Moreover, the prevalence of anxiety disorders is much higher in adolescents than in adults [6], reflecting the vulnerability to the onset of certain types of psychopathology during adolescence [7]. It is believed that adolescence is a sensitive period for the development of mental illnesses due to some aberrations that can occur during typical adolescent brain maturation [8], together with some behavioral characteristics of adolescence, such as increasingly powerful emotional responses to social stimuli, increased stress susceptibility, and risk/reward appraisal and motivation [9, 10].

Although there is a large group of effective drugs for the treatment of depression in adults, currently, only selective serotonin reuptake inhibitors (SSRIs), specifically two of them, fluoxetine and escitalopram, have been approved by the Food and Drug Administration (FDA) to treat pediatric MDD. According to the chemical imbalance and serotonin theories of depression, pharmacological and therapeutic effects of SSRIs, e.g., fluoxetine, are related to a correction of prior chemical imbalance in neurotransmitters levels [11]. However, recently, these theories of depression, and thus the mechanisms of SSRIs action have been called into question [11, 12]. It has been postulated that the therapeutic effects of fluoxetine may be rather mediated by its influence on neuroplasticity by affecting gene expression, inducing epigenetic changes and modifying synaptic transmission through, e.g., synaptic remodeling or long-term potentiation/depression [11, 13]. In the context of pharmacotherapy of young patients it is worth emphasising that the developing brain has broad ability and sensitivity to neuroplastic changes [14]. Fluoxetine is considered the first-choice drug in the pharmacotherapy of MDD and anxiety disorders in children and adolescents. It is also registered in obsessive–compulsive disorder (OCD) in pediatric population and in anxiety disorders such as panic disorder in adults [15]. Fluoxetine and escitalopram are registered in MDD patients older than 8 and 12 years old, respectively [16]. Nevertheless, in late 2004, the FDA issued a “black box” warning on antidepressants, indicating their association with an increased risk of suicidal thoughts and behavior in children and adolescents [17], reported by preceding and subsequent meta-analyses assessing antidepressant use in the pediatric population [18–22]. Although fluoxetine favorable risk–benefit profile in adolescents with MDD has been reported [23–25], the developing brain is sensitive to pharmacological interventions, and chronic fluoxetine treatment could influence the maturation and plasticity of the brain since serotonin is pivotal in the regulation of brain development during adolescence. 5-HT promotes cellular mitosis, migration and maturation of neurons and glial cells [26]. During the adolescent period, reorganization of serotoninergic innervation patterns, changes in 5-HT receptor expression, and a steady increase in serotonin transporters have been reported. It has been suggested that biochemical and morphological development of 5-HTergic function reaches a maximal peak during adolescence at postnatal days (PD) 35–45 in rats [27–30]. Notably, remodeling of the 5-HT system during adolescence is most pronounced in the frontal and limbic areas, which are involved in cognitive behaviors, motivation, emotion and memory [9].

Some animal research has shown age-specific effects of fluoxetine exposure. Early (starting at PD 25) but not later (starting around PD 60) fluoxetine treatment significantly increased serotonin transporter densities in the rodent frontal cortex [31, 32], suggesting a stimulatory effect of early adolescence fluoxetine on the outgrowth of serotonergic synaptic terminals. Adolescent fluoxetine-induced upregulation of serotonin transporters in the neocortex and hippocampus was also reported in nonhuman primates [33]. These effects might be mediated by serotonin-triggered activation of 5-HT-1A receptors on neighboring astrocytes and, consequently, the release of the neurotrophic peptide S-100β, which promotes serotonergic hyperinnervation [34]. Furthermore, as recently shown, adolescent modulation of the 5-HT system through the blockade of 5-HT-1A receptors resulted in increased anxiety in adulthood. Interestingly, anxiety-like behavior was observed only when 5-HT-1A receptor signaling was blocked between PD 35–50 but not at the later timepoint [35]. The above data suggest that the adolescence period is sensitive to changes in 5-HT signaling and that chronic SSRIs during this period of time could alter the maturation of serotonin neurotransmission and/or affect developmental neuroplasticity, resulting in changes in emotion-related behavior.

Unfortunately, SSRI treatment approved for adolescents has largely been studied in adult animal and human subjects and might not adequately reflect the influence of these drugs on the developing brain. Indeed, the drug effects observed in adolescence could differ from those in adulthood due to interactions with developmental changes appearing in immature brains [36]. Additionally, human studies conducted in the pediatric population are limited for ethical reasons. Thus, prior to and in addition to examination in humans, the effects of SSRIs in adolescents should be investigated in animal models. However, animal studies assessing behavioral consequences resulting from adolescent SSRI exposure have been rather ambiguous. Given the lack of comprehensive and conclusive information from animal studies regarding adolescent treatment with the most commonly prescribed antidepressant in the human pediatric population, fluoxetine, we aimed to summarize the behavioral responsivity of adolescent rodents to chronic fluoxetine exposure. Specifically, the evaluation and summarization of all studies reporting the effects of chronic adolescent fluoxetine on anxiety- and depressive-like behaviors in adolescents were performed following a systemic review and meta-analysis. To give a more comprehensive picture of adolescent fluoxetine exposure, we included and analyzed separately data from naïve animals and those subjected to chronic stress procedure during their lifetime. Chronic stress exposure is an important risk factor contributing to the development of psychiatric disorders and numerous animal models based on chronic stress paradigms, such as chronic restraint stress [37], chronic social defeat stress [38], chronic unpredictable mild stress [39, 40] and early maternal separation stress [41] are applied to study depressive- and anxiety-like symptoms.

According to the PICO (Population, Intervention, Comparison and Outcome) approaches, inclusion criteria were established as follows: Population: laboratory rats or mice of any sex; Intervention: chronic fluoxetine given during adolescence; Comparison: fluoxetine vs. control group receiving vehicle; Outcome: anxiety-like behavior and depression-like behavior measured during adolescence. To analyze potential sources of the results heterogeneity we pre-planned to perform subgroup analyses for the sex of animals and rodent species as sex differences and genetic background may have an important impact on the clinical presentation of measured effects.

## Materials and methods

### Literature search strategy

A systematic screening was performed using the following databases: Medline (via PubMed), Web of Science Core Collection, and ScienceDirect until April 12, 2022. The search strategy was based on the MeSH (medical subject heading) terms and Emtree, combined with Boole’s logical operators with the major search terms “fluoxetine” AND “adolescence” (Online Resource ESM_1) and supplemented with hand-searching of the reference lists of identified studies. Identified meta-analyses and systematic reviews were also searched for relevant data.

### Selection criteria

Two independent reviewers (J.K. and A.C.) used the same search strategy to identify relevant research articles, starting with the title and abstract and followed by a full-text screen. All disagreements were resolved through discussion to reach consensus. The study selection was based on the titles and abstracts and, finally, on the full-text articles. The meta-analysis included preclinical studies comparing chronic fluoxetine administration with a vehicle during adolescence in rodents. The systematic review protocol was registered at the International prospective register of systematic reviews (PROSPERO) under number CRD42022307973. According to the CAMARADES guidelines for meta-analyses of preclinical studies [42], a predefined study inclusion was as follow: (1) controlled studies in laboratory rats or mice (both sexes included); (2) naïve or exposed to chronic stress procedure animals; (3) chronic fluoxetine (at least 10 days) irrespective of the route of administration and dose of the drug; (4) fluoxetine given during the period of adolescence (PD 20–60); (5) vehicle as a comparator; (6) evaluation of fluoxetine effects in behavioral models assessing anxiety-like behavior (primary end-point—time in open arms of an elevated plus maze—EPM) or depression-like behavior (immobility time in the forced swim test—FST), if possible, locomotor activity measured in the open field (OF) test was planned to be included, as basal locomotor activity can interfere with measures in other behavioral tests; (7) behavioral outcomes measured during adolescence (until PD 60 in mice and PD 70 in rats; timeframe of adolescence in rodents was suggested to last until PD 60 [43]; however, recent papers have postulated that rodent adolescence lasts until PD 60 in mice and PD 70 in rats [44, 45]) and (8) English-language, peer-reviewed publications. Safety measures were not included as an outcome because they were not analyzed in the original studies. The general assumption is that in animal studies assessing anxiety- and depressive-like or other behaviors, the dose of the drug used should be safe, otherwise its toxicity would affect animal welfare and interfere with the obtained results and the main research hypothesis. Studies were excluded based on the following criteria: (1) no access to the full text; (2) acute or subchronic (a few days) fluoxetine administration; (3) no behavioral outcomes of interest reported; (4) behavioral outcomes measured in adulthood; (5) studies including knockout animals only; (6) previous exposure of animals to any other drug or procedure other than chronic stress exposure, unless results from untreated animals serving as controls were reported; and (7) means, standard deviations and sample sizes unavailable for the outcomes measured. Data reported only in abstract form (with no associated full text) were rejected due to the lack of detailed information about methodology, animal subjects, and results. Case studies and studies without a control group were excluded. All full-text primary studies fulfilling pre-defined criteria were included in the qualitative synthesis.

### Data extraction and outcome measures

Data extraction and calculations were done independently by two reviewers (J.K.) (I.M.M.). In case of disagreement between the two researchers, a third reviewer (A. C.) was consulted. According to CAMARADES guidelines and predefined protocol, extracted data included study design and sample characteristics, details of interventions and regimens and outcomes data. If an additional drug or procedure other than chronic stress exposure was applied in the study, only data from the control groups (without additional interventions) were extracted. One specific effect size from the single behavioral test common for included studies was used in the meta-analysis. Multiple subgroups within a single study (e.g., sex of animals or different rodent strains) were included as independent standardized mean difference (SMD), provided that each treatment group had a separate matched control group. If the number of animals in the group was reported as a range (e.g., 8–10; 9–10), the middle or the lowest number of animals per group was used for the meta-analysis. When results were available only in graphical format, data were extracted using WebPlotDigitizer graph digitization software [46] recommended for use in systematic reviews [47]. Missing data were requested from the corresponding authors of the primary studies.

### Data analysis

Potential sources of bias were identified for each trial using SYRCLE’s tool for assessing the risk of bias in animal studies [48]. Risk of bias assessment was conducted by one reviewer (J.K.) based on ten items related to selection bias (three items), performance bias (two items), detection bias (two items), attrition bias, reporting bias and other biases (one item each). To evaluate selection bias, the propriety of allocation sequence generation and concealing, as well as the similarity of baseline characteristics between groups were assessed. Evaluation of performance bias was performed by assessment of the blinding of the investigators and random housing of the animals during the experiment (whether the authors randomly place the cages or animals within the room). Random selection of animals for outcome assessment and blinding of the assessors were used for evaluation of detection bias. Low risk of attrition bias was evaluated when an adequate description of reasons for missing data or exclusion of the animals from the outcome assessment was reported. Item regarding the assessment of selective outcome reporting was used to assess reporting bias. Among the tenth item other sources of biases were evaluated (e.g. discrepancies in common data reporting in the main text and figures of the primary study).

Funnel plots were generated to assess publication bias. For continuous outcomes, the SMDs between fluoxetine and the comparator with 95% confidence intervals (CIs) were calculated. Statistical heterogeneity among studies and subgroups was evaluated with the Chi^2^ and I^2^ tests. The I^2^ values of 0%, 25%, 50% and 75% were estimated as “no”, “low”, “moderate” and “high” heterogeneity, respectively [49]. The random effects model was applied because it has greater generalizability for empirical examination of summary effect measures in meta-analyses [50]. Statistical significance was defined at a *p* value of less than 0.05. The results were presented as forest plots using the Review Manager Software package, version 5.4.1 (the Cochrane collaboration).

Naïve and exposed to stress animals were meta-analyzed separately, according to the protocol. Subgroup analyses were pre-planned for the sex of animals and rodent species (rats vs. mice), while the decision to perform the subgroup analyses of the dose of fluoxetine was made post-hoc. Subgroup analyses were planned to analyze the sources of heterogeneity. Pre-planned sensitivity analysis was scheduled as leave-one-study-out and was performed to assess how each individual study affects the overall estimate of the rest of the studies.

## Results

### Search results

**Fig. 1:**
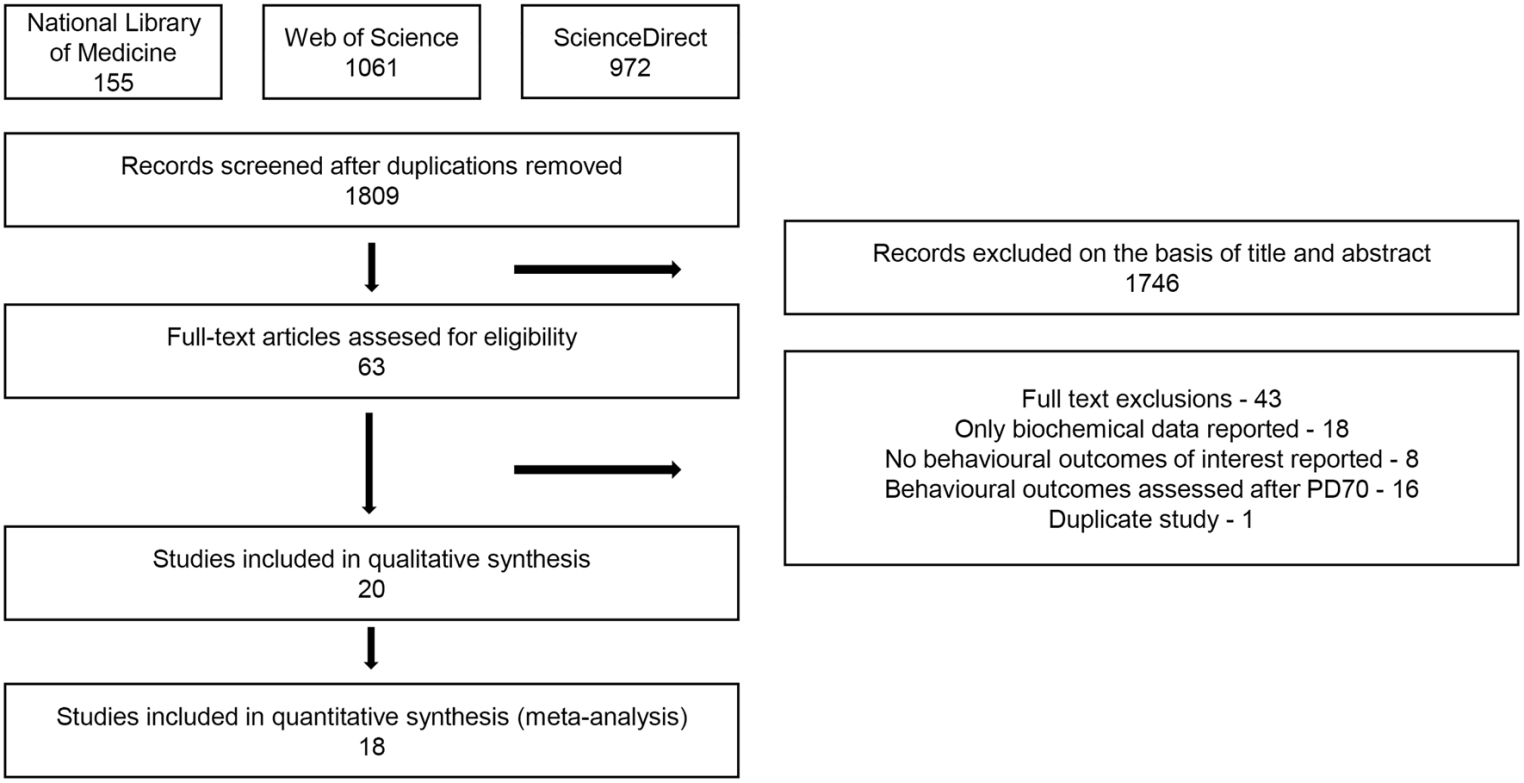
Flow diagram of study identification and selection process according to PRISMA guidelines. *PD* postnatal day

A systematic literature search identified 1809 items after duplicates were removed. The selection of titles and abstracts resulted in 63 potentially relevant articles, of which 43 were excluded due to the reasons presented in Fig. 1. Finally, 20 studies [32, 51–69] met the predefined inclusion criteria and were included in the qualitative synthesis. Of the 20 included studies, 18 trials were suitable for quantitative synthesis (meta-analysis). As in two trials no data available for meta-analyses were reported, they were included in qualitative synthesis only [58, 66]. A detailed flow diagram of the publication selection process is shown in Fig. 1. The methodology and main characteristics of the included studies and reported outcomes are described in Tables 1 and 2. Raw data extracted from primary studies are presented in Online Resource ESM_2. There was heterogeneity in the fluoxetine schedules between studies. Drugs were administered intraperitoneally (*IP*), subcutaneously (*SC)*, orally (in drinking water or by gavage) or via minipumps in a broad range of doses (3–25 mg/kg/day). Therefore, for the main meta-analyses, the middle value of the dose range (10 mg/kg/day) was considered, if provided. Fluoxetine was administered for at least 10 days starting at PD 20–35 (except Oh et al.’s study [51], in which fluoxetine was initiated at PD 14). Although inclusion of the Oh et al.’s study [51] is a limitation of our review, we finally decided to include it in the meta-analysis as fluoxetine exposure lasted during 3 full weeks of adolescence period. All [32, 51–56, 60–65, 68, 69], but four studies [57, 59, 66, 67] reported using only male rodents. Most studies used rats, while only two studies included mouse subjects [51, 66]. In most studies, either any or a very short (24 h) drug-free interval was applied, while in three studies, tests were conducted approximately 7 [32], 10 [53], and 26 [61] days after treatment discontinuation. Anxiety-like behavior was assessed using the EPM, OF testing, latency to feed, light/dark box and startle magnitude. Depression-like behavior was assessed using the FST, sucrose preference, tail suspension, cocaine place preference (CPP) and splashed test (Tables 1 and 2). According to the pre-defined protocol, meta-analyses were performed based on data from the EPM and FST as these tests are the most frequently used for measuring anxiety- and depressive-like behavior in rodents, and supplemented with meta-analyses based on OF data to analyze influence of fluoxetine treatment on a basal locomotor activity. Stress-exposed animals were either subjected to neonatal maternal separation (during the first two weeks of life) [57, 60, 64, 65, 68], adolescent social isolation [63] plus social defeat procedure [58] or subthreshold chronic unpredictable mild stress paradigm (SCUMS) [69] (Table 2).

**Table 1:** Characteristics of studies included in the qualitative analysis regarding naïve animals (not subjected to chronic stress exposure during the lifetime)

Author, Year	Country	Species and sex	Fluoxetine regimen*	Age of fluoxetine treatment (only adolescent treatment period)	Tests	Age of assessment (*N* per group)	Outcomes#
*Studies included in quantitative synthesis (meta-analysis)*							
Oh 2009 [51]	US	Male C57Bl/6 and Swiss Webster (SW) mice	PD 14–21—minipumps (2, 3, 4 mg/kg), PD 22–45 – 0.015 and 0.03 mg/ml oral in drinking water (~ 1.5 i 3 mg/kg)^	PD 14 ~ 45 (2–6.5 week)	EPM, 3 mg/kg	PD 42 SW (29–31) C57 (34–36)	SW: ↔ Time in open arms
							C57: ↓ Time in open arms
					FST, 3 mg/kg	PD 42 SW (29–31) C57 (34–36)	SW: ↔ Immobility time
							C57: ↔ Immobility time
					OF, 3 mg/kg	PD 42 SW (29–31) C57 (34–36)	SW: ↔ Center time
							C57: ↓ Center time ↓ Total activity
					NIH, 3 mg/kg	PD 42 SW (29–31) C57 (34–36)	SW: ↑ Latency (novel cage); ↔ latency (home cage)
							C57: ↑ Latency (novel cage); ↔ latency (home cage)
Iñiguez 2010 [52]	US	Male Sprague–Dawley rats	10 mg/kg, twice daily, *IP*	PD 35–49	EPM	PD 50 (8)	↓ Time in open arms; ↓ open arm entries
					FST	PD 50 (5–6)	↑ Latency to immobility ↓ Immobility time ↑ Swim count ↑ Climbing count ↓ Float count
					OF	PD 50 (10)	↔ Distance travelled
					Sucrose preference	PD 50 (13)	Sucrose preference: ↔ (0.125% sucrose concentration; 0.5%; 1.0%); ↑(0.25%) Total liquid intake: ↔ (0.125%; 0.25%; 0.5%; 1.0%)
					Novel object approach in a familiar environment	PD 50 (9–10)	↑ Latency to approach ↑ Time spent with object
					Food approach in a novel environment	PD 50 (9)	↑ Latency to feed
Homberg 2011 [53]	The Netherlands	Male Wistar Unilever rats	12 mg/kg, pills by oral gavage	PD 25–49	EPM	PD 59 (10)	↓ Time in open arms
					FST	PD 59 (10)	↑ Immobility time
					OF	PD 56 (10)	↔ Distance travelled
Vorhees 2011 [54]	US	Male Sprague–Dawley rats	3, 10 mg/kg, oral by gavage	PD 33–62	EPM	PD 57 (20)	Time in open arms: ↔ (3 and 10 mg/kg) Latency to first open arm entry: ↔ (3 and 10 mg/kg) Open arm head dips: ↔ (3 and 10 mg/kg) Open arm entries: ↔ (3 and 10 mg/kg)
					FST	PD 61–62 (19–21)	Immobility time: ↔ (3 and 10 mg/kg)
Warren 2011 [55]	US	Male Sprague–Dawley rats	2.5 mg/kg, twice daily, *IP*	PD 20–34	EPM	PD 35 (7–8)	↔ Time in open arms
					FST	PD 35 (7–8)	↑ Latency to immobility ↓ Immobility time ↓ Immobility count ↑ Swim count ↑ Climbing count
					CPC	PD 35 (5–9)	Place conditioning: ↔ (5 mg/kg); ↑(10 mg/kg)
Bouet 2012 [32]	French, the Netherlands and UK	Male Wistar rats, only control groups without acute challenge of FLX analysed	5 mg/kg, oral by gavage	PD 25–46	EPM	PD 53 (10)	↔ Open arm entries
					FST	PD 53 (10)	↔ Immobility time
					OF	PD 53 (10)	↔ Horizontal activity (number of squares crossed)
Sass 2012 [56]	Denmark	Male Wistar rats	10 mg/kg, *IP*	PD 28–60	OF	PD 55–56 (12)	↓ Distance ↓ Number of entries into center
Yoo 2013 [57]	Republic of Korea	Female Sprague–Dawley rats, only control groups without previous MS analysed	10 mg/kg, *IP*	PD 35–54	EPM	PD 54 (5–6)	↓Time in open arms ↑ Time in closed arms
					FST	PD 46 (5–6)	Duration of: ↑ Immobility ↔ Swimming ↓ Struggling
					OF	PD 44 (5–6)	↔ Activity counts ↔ Distance travelled
Amodeo 2015 [59]	US	Male and female Sprague–Dawley rats	5, 10, 20 mg/kg, *IP*	PD 35–44	EPM	PD 45 (9–11) results reported only for 10 mg/kg	♂ and ♀: Time in open arms: ↔ (dose 5 and 10 mg/kg); ↓ (dose 20 mg/kg) ♂ and ♀: Head dips: ↔ (dose 5 and 10 mg/kg); ↓ (dose 20 mg/kg) ♂ and ♀: Closed arm entries: ↔ (dose 5 and 10 mg/kg); ↓ (dose 20 mg/kg)
					Sucrose preference	PD 45 (11–12) results reported only for 10 mg/kg	♂ and ♀**:** ↔ Sucrose preference (2.0% sucrose concentration)
					LD box	PD 45 (9–11)	♂ and ♀**:** Time in light compartment: ↔ (dose 5 mg/kg); ↓ (dose 10 and 20 mg/kg) ♂ and ♀**:** Light/dark transitions: ↔ (dose 5; 10 and 20 mg/kg)
					Acustic startle	PD 45 (9–11)	♂ and ♀**:** Startle magnitude: ↔ (dose 5; 10 and 20 mg/kg); ♂ and ♀**:** Startle habituation: ↔ (dose 5; 10 and 20 mg/kg)
Badenhorst 2017 [61]	South Africa	Male FSL rats	10 mg/kg, *SC*	PD 21–34	FST	PD 60 (15)	↔ Immobility time
					OF	PD 60 (15)	↓ Lines crossed ↔ Time spent in the center
Schoeman 2017 [62]	South Africa	Male FSL rats, only control groups without additional exercise intensities analysed	5 or 10 mg/kg, *SC*	PD 21–34	FST	PD 35 (7–8)	Immobility time: ↓ 5 mg/kg; ↔ 10 mg/kg; Climbing: ↑ 5 mg/kg; ↔ 10 mg/kg; Swimming: ↔ 5 and 10 mg/kg
						PD 60 (12), results reported only for 5 mg/kg	↓ Immobility time ↑ Climbing ↔ Swimming
					OF	PD 35 (7–8)	Line crossings: ↔ 5 mg/kg; ↓ 10 mg/kg
						PD 60 (12), results reported only for 5 mg/kg	↔ Line crossings
Sonei 2017 [63]	Iran, Canada	Male Wistar Albino rats, only control groups without social isolation analysed	7.5 mg/kg oral in drinking water	PD 28—49	EPM	PD 50 (8)	↓ Time in open arms ↓ Open arm entries
					FST	PD 50 (8)	↔ Immobility time
					OF	PD 50 (8)	↔ Vertical activity ↔ Horizontal activity (number of squares crossed)
					Splashed test	PD 50 + (6)	↔ First latency ↔ Grooming time
Masrour 2018 [65]	Iran	Male Albino Wistar rats, only control groups without MS analysed	5 mg/kg, *IP*	PD 30–60	FST	PD 60 (6–8)	↔ Immobility time
					OF	PD 60 (6–8)	↔ Horizontal activity (number of squares crossed)
					Sucrose preference test	PD 60 (6–8)	↔ Sucrose consumption (1,0% sucrose concentration)
					Splashed test	PD 60 (6–8)	↔ Grooming time
Sahafi 2018 [64]	Iran	Male Albino Wistar rats, only control groups without previous MS analysed	5 mg/kg, *IP*	PD 30–60	FST	PD 60 (6)	↔ Immobility time
					OF	PD 60 (6)	↔ Horizontal activity (number of squares crossed)
					Sucrose preference test	PD 60 (6)	↔ Sucrose consumption (1,0% sucrose concentration)
					Splash test	PD 60 (6)	↔ Grooming activity time
Sadegzadeh 2020 [67]	Iran	Male and female Wistar rats	5 mg/kg, oral by gavage	PD 21–60	EPM	PD 64 (8)	Time in open arms: ↔ ♂; ↑ ♀; Open arm entries: ↔ ♂, ♀; Locomotor activity: ↔ ♂; ↑ ♀
					OF	PD 61 (8)	Number of rearing: ↔ ♂; ↑ ♀; Number of grooming: ↔ ♂, ♀; Distance travelled: ↔ ♂; ↑ ♀; Time spent in inner zone: ↑ ♂; ↔ ♀; Number of entries to inner zone: ↔ ♂; ↑ ♀; Number of fecal boli: ↔ ♂, ♀
Zolfaghari 2021 [68]	Iran, Canada	Male Albino Wistar rats, only groups without previous MS analysed	5 mg/kg, *IP*	PD 28–60	OF	PD 61 (7)	↔ Horizontal activity (number of squares crossed) ↔ Number of rearings ↓ Time spent in the center
					FST	PD 63 (7)	↔ Immobility time
					EPM	PD 65 (7)	↓ Time in open arms ↓ Open arm entries
					Sucrose preference test	PD 70 (7)	↔ Sucrose consumption (1,0% sucrose concentration)
Yu 2022 [69]	China	Male Sprague–Dawley rats, only groups without previous SCUMS analysed	10 mg/kg, twice daily, *IP*	PD 24–38	FST	PD 44 (12)	↔ Immobility time
					NSFT	PD 42 (12)	↔ Latency to feed ↔ Food consumption
					Sucrose preference test	PD 40 (12)	↔ Sucrose consumption (1,0% sucrose concentration)
*Studies included in qualitative synthesis*							
Flores-Ramirez 2018 [66]	US	Female C57Bl/6 mice	250 mg/l, oral in drinking water (~ 25 mg/kg)	PD 35–49	Tail suspension	PD 50 (10)	↑ Latency to immobility ↓ Total immobility

*Dose per day; ^ results were reported only for a dose of 3 mg/kg; # outcomes assessing anxiety-like, depressive-like and rewarding effects included; arrows indicate statistically significant increase (↑), decrease (↓) or no change ( ↔) in FLX group vs vehicle group

Note: Some trials evaluated additional treatment arms, time points of assessment or outcome measures not listed here that were outside of the scope of the eligibility criteria for this analysis

*CPC* cocaine place conditioning, *EPM *elevated plus maze, *FLX *fluoxetine, *FSL *Flinder sensitive line, *FST *forced swim test, *IP *intraperitoneally, *LD *light–dark box, *MS *maternal separation, *NIH* novelty-induced hypophagia, *NSFT *novelty-suppressed feeding test and food consumption test, *OF *open field, *PD *postnatal day, *SC* subcutaneous, *SCUMS *subthreshold chronic unpredictable mild stress paradigm, *SW *Swiss Webster

**Table 2:** Characteristics of studies included in the qualitative analysis regarding animals subjected to chronic stress exposure during the lifetime

Author, Year	Country	Species and sex	Stress procedure applied	Fluoxetine regimen*	Age of fluoxetine treatment (only adolescent treatment period)	Tests	Age of assessment (*N* per group)	Outcomes#
*Studies included in quantitative synthesis (meta-analysis)*								
Yoo 2013 [57]	Republic of Korea	Female Sprague–Dawley rats, only groups with previous MS analysed	Maternal separation for 3 h daily from PD 1–14	10 mg/kg, *IP*	PD 35–54	EPM	PD 54 (5–6)	↓Time in open arm ↑ Time in closed arm
						FST	PD 46 (5–6)	Duration of: ↓ Immobility ↑ Swimming ↓ Struggling
						OF	PD 44 (5–6)	↔ Activity counts ↔ Distance travelled
Sadeghi 2016 [60]	Iran	Male Albino Wistar rats, only groups with previous MS analysed	Maternal separation for 3 h daily from PD 2–14	5 mg/kg, *IP*	PD 28–60	OF	PD 61 (8)	↔ Horizontal activity (number of squares crossed)
						Splashed test	PD 62 (8)	↑ Grooming time
						Sucrose preference test	PD 65 (8)	↔ Sucrose consumption (1,0% sucrose concentration)
						FST	PD 66 (8)	↓ Immobility time
Sonei 2017 [63]	Iran, Canada	Male Wistar Albino rats, only groups with social isolation analysed	Individually housed from PD 21–49	7.5 mg/kg oral in drinking water	PD 28–49	EPM	PD 50 (8)	↔ Time spent in open arms ↔ Open arm entries
						FST	PD 50 (8)	↓ Immobility time
						OF	PD 50 (8)	↓ Vertical activity ↓ Horizontal activity (number of squares crossed)
						Splashed test	PD50 + (6)	↓ First latency ↑ Grooming time
Masrour 2018 [65]	Iran	Male Albino Wistar rats, only control groups without MS analysed	Maternal separation for 3 h daily from PD 2–14	5 mg/kg, *IP*	PD 30–60	FST	PD 60 (6–8)	↓ Immobility
						OF	PD 60 (6–8)	↔ Horizontal activity (number of squares crossed)
						Sucrose preference test	PD 60 (6–8)	↔ Sucrose consumption (1,0% sucrose concentration)
						Splashed test	PD 60 (6–8)	↑ Grooming time
Sahafi 2018 [64]	Iran	Male Albino Wistar rats, only groups with previous MS analysed	Maternal separation for 3 h daily from PD 2–14	5 mg/kg, *IP*	PD 30–60	FST	PD 60 (6)	↓ Immobility
						OF	PD 60 (6)	↔ Horizontal activity (number of squares crossed)
						Sucrose preference test	PD 60 (6)	↔ Sucrose consumption (1,0% sucrose concentration)
						Splash test	PD 60 (6)	↑ Grooming activity time
Zolfaghari 2021 [68]	Iran, Canada	Male Albino Wistar rats, only groups with previous MS analysed	Maternal separation for 3 h daily from PD 2–14	5 mg/kg, *IP*	PD 28–60	OF	PD 61 (7)	↔ Horizontal activity (number of squares crossed) ↔ Number of rearings ↓ Time spent in the center
						FST	PD 63 (7)	↓ Immobility time
						EPM	PD 65 (7)	↔ Time in open arms ↔ Open arm entries
						Sucrose preference test	PD 70 (7)	↑ Sucrose consumption (1,0% sucrose concentration)
Yu 2022 [69]	China	Male Sprague–Dawley rats, only groups with previous SCUMS analysed	Subthreshold chronic unpredictable mild stress from PD 29–38	10 mg/kg, twice daily, *IP*	PD 24–38	FST	PD 44 (12)	↓ Immobility time
						NSFT	PD 42 (12)	↓ Latency to feed ↔ Food consumption
						Sucrose preference test	PD 40 (12)	↑ Sucrose consumption (1,0% sucrose concentration)
*Studies included in qualitative synthesis*								
Bourke 2014 [58]	USA	Male Wistar rats	Individually housed and exposed daily to social defeat from PD 28–50	12 mg/kg, oral by gavage	PD 28–50	FST	PD 53 (6–8)	↔ Latency to immobility
						Sucrose preference test	PD 52 (6–8)	↔ Sucrose consumption (0,8% sucrose concentration)

*Dose per day; ^#^outcomes assessing anxiety-like, depressive-like and rewarding effects included; arrows indicate statistically significant increase (↑), decrease (↓) or no change ( ↔) in FLX group vs vehicle group

Note: Some trials evaluated additional treatment arms, time points of assessment or outcome measures not listed here that were outside of the scope of the eligibility criteria for this analysis

*EPM* elevated plus maze, *FLX* fluoxetine, *FST* forced swim test, *IP* intraperitoneally, *MS* maternal separation, *NSFT* novelty-suppressed feeding test and food consumption test, *OF* open field, *PD* postnatal day, *SCUMS* subthreshold chronic unpredictable mild stress paradigm

### Studies excluded from the qualitative synthesis

From 63 full-text articles assessed for eligibility 43 studies were excluded. Among studies reporting behavioral outcomes, in twelve trials behavioral measures were reported for early adulthood (after PD 60—mice, or after PD 70—rats) [70–81], while in four trials, behavioral measures were reported for later (after PD 100) adulthood [82–85]. Eight studies did not report behavioral outcomes of interest or provided data as a statistical analysis only [86–93]. One study was a duplicate study [94]. Eighteen studies reported only biochemical data [95–112].

### Risk of bias assessment

Potential sources of bias are summarized in Table 3. Risk of selection bias was unclear for all included studies due to incomplete information about the approach to the allocation of study subjects to treatment groups and the baseline characteristics for animals in each of the treatment groups. Performance bias regarding random housing of animals and detection bias regarding random outcome assessment was unclear for all included studies. Visual inspection of the funnel plots addressing publication bias of studies included in particular meta-analyses regarding naïve animals showed relatively symmetrical distribution (Online Resource ESM_3). Too few studies included in meta-analyses regarding stress-exposed animals prevented the evaluation of publication bias.

**Table 3:**
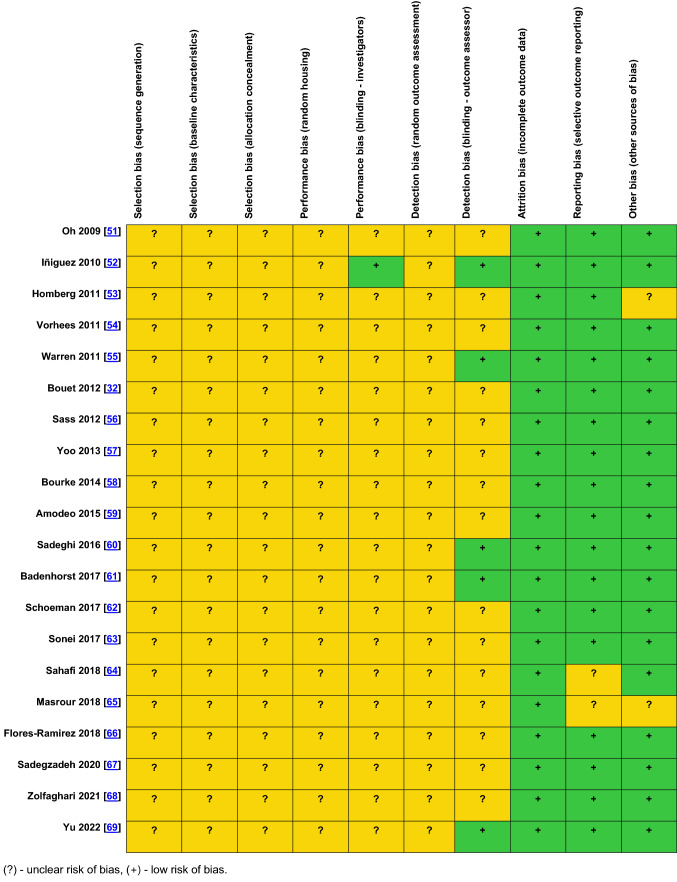
SYRCLE’ tool for assessing a risk of bias

### Anxiety-like behavior

#### Naïve animals

**Fig. 2:**
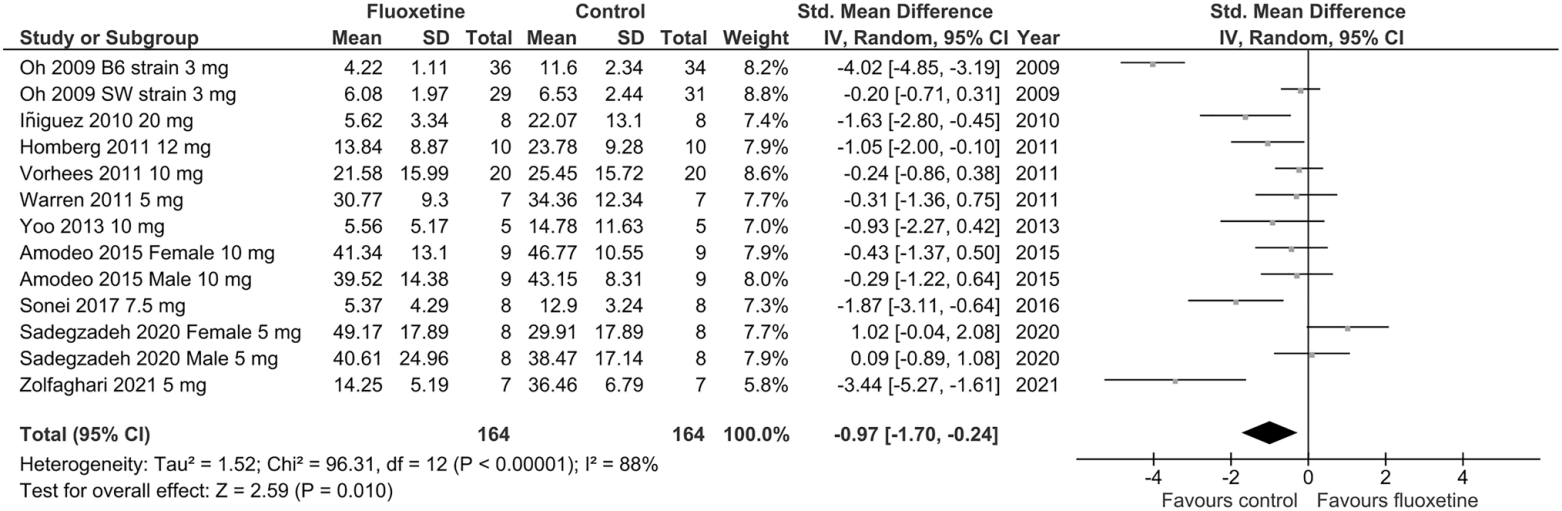
Forest plot of the effects of chronic fluoxetine at doses of 3–20 mg/kg/day vs. vehicle on anxiety-like behavior in naïve animals measured as time spent in the open arms of the EPM

Ten trials assessed anxiety-like behavior using a measure of time spent in the open arms of the EPM in naïve rodents. Two studies provided separate results for male and female subjects [59, 67], while one study reported separate results for two different mouse strains [51]. Separate results for different doses of fluoxetine were reported in two studies [54, 59]. As shown in Fig. 2, chronic fluoxetine exposure during adolescence significantly decreased the time spent in the open arms of the EPM compared to vehicle (SMD =  – 0.97; *p* = 0.01; test for heterogeneity: Chi^2^ = 96.31; df = 12; *p* < 0.00001; *I*^2^ = 88%). Sensitivity study analyses showed that the effect remained unchanged in leave-one-out analyses. However, after excluding individual studies [52, 53, 63, 68], the level of SMD significance decreased (*p* = 0.02; *p* = 0.03) (Online Resource ESM_4). In three of the fourth studies [52, 53, 63] fluoxetine was administered in relatively high doses (≥ 7,5 mg/kg). To further analyze the effect of fluoxetine dose on anxiety-like effects, an exploratory analysis was performed. Pooled data of fluoxetine administered at higher doses (7.5–20 mg/kg/day) showed a significant decrease in time spent in the open arms of the EPM vs. vehicle group (SMD =  – 0.79; *p* = 0.0009; test for heterogeneity: Chi^2^ = 9.64; df = 6; *p* = 0.14; *I*^2^ = 38%) (Online Resource ESM_5). Meta-analysis of the effects of lower fluoxetine doses (3–5 mg/kg/day) showed no significant differences between the drug and vehicle groups regarding time spent in the open arms of the EPM (SMD =  – 0.70 [95% CI  – 1.68; 0.29]; *p* = 0.16; test for heterogeneity: Chi^2^ = 93.98; df = 8; *p* < 0.00001; *I*^2^ = 91%) (Online Resource ESM_5). Leave-one-out analyses showed no marked difference in meta-analyses conducted for lower and higher fluoxetine doses, suggesting that they were not driven by one single study. Although the test for subgroup differences indicated that there was no statistically significant subgroup effect (Online Resource ESM_5), a smaller number of studies and animals contributed data to the higher dose than to the lower dose subgroup, meaning that the analysis may not be able to detect subgroup differences [113].

**Fig. 3:**
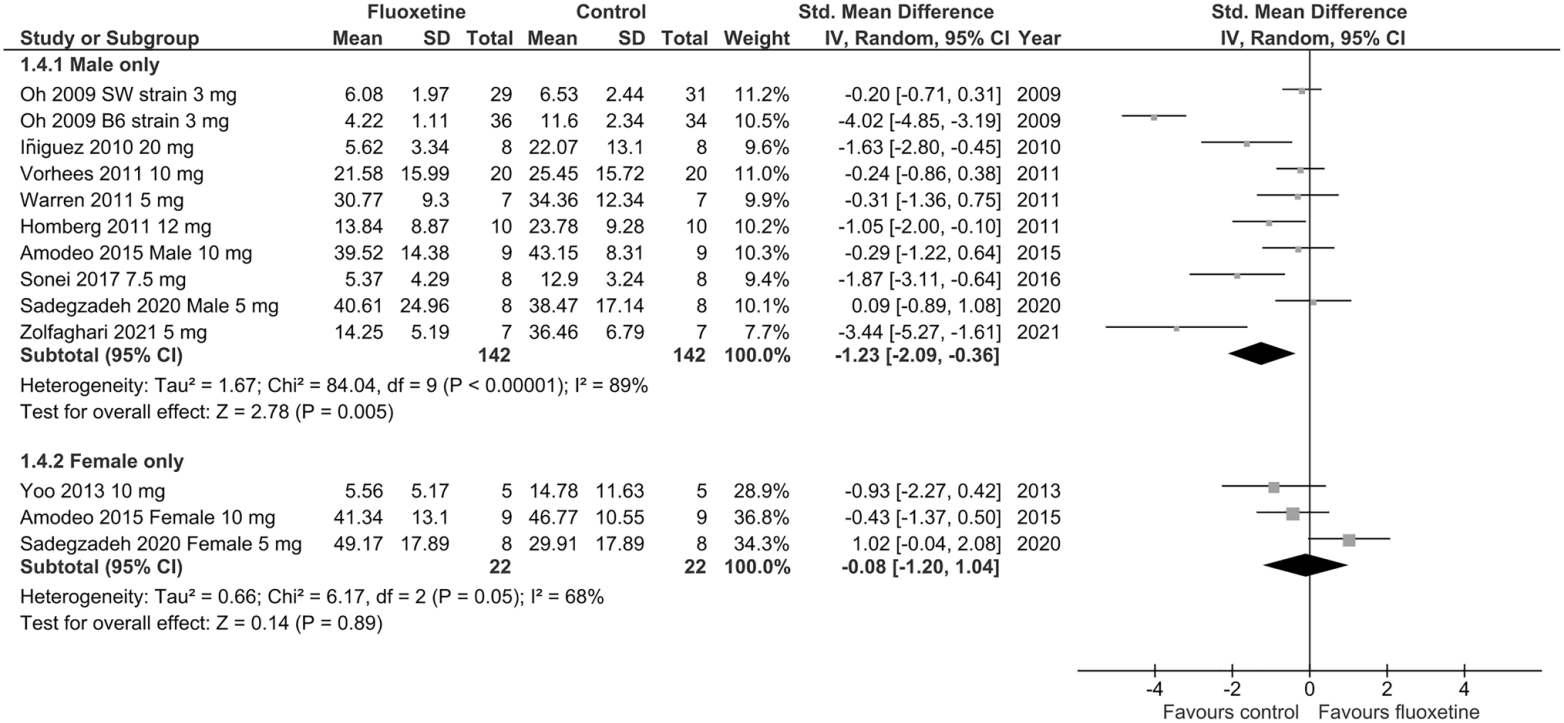
Forest plot of the effects of chronic fluoxetine at doses of 3–20 mg/kg/day vs. vehicle on anxiety-like behavior in naïve males and females measured as time spent in the open arms of the EPM

Sex effects were also assessed in predefined subgroup analyses. Pooled data from eight studies showed that fluoxetine-induced anxiety-like behavior was significant in males (SMD =  – 1.23; *p* = 0.005; test for heterogeneity: Chi^2^ = 84.04; df = 9; *p* < 0.00001; *I*^2^ = 89%) (Fig. 3), while no significant differences in time spent in the open arms of the EPM between fluoxetine and vehicle were reported in females (SMD =  – 0.08; *p* = 0.89; test for heterogeneity: Chi^2^ = 6.17; df = 2; *p* = 0.05; *I*^2^ = 68%) (Fig. 3). However, conclusions from the latter comparison were strongly limited since it was based on 3 studies only. Since all but one study [51] included only rat subjects, it was not possible to analyze species effects. There was moderate to high heterogeneity (*I*^2^ > 50%) across the studies included in particular meta-analyses regarding time spent in the open arms of the EPM in naïve animals, while the heterogeneity of intervention effects was low (*I*^2^ > 38%) only in the meta-analysis regarding higher doses of fluoxetine.

**Fig. 4:**
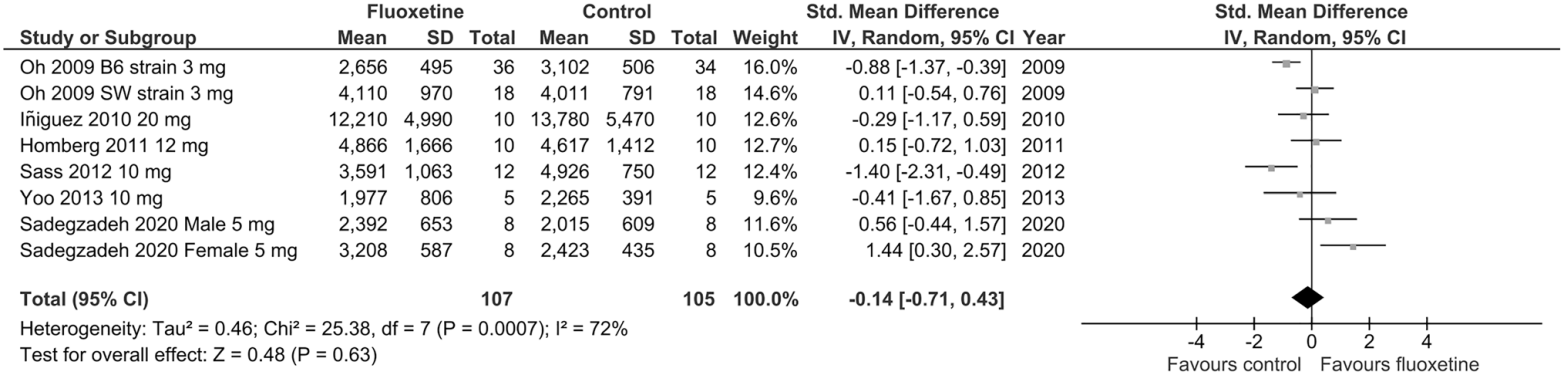
Forest plot of the effects of chronic fluoxetine at doses of 3–20 mg/kg/day vs. vehicle on locomotor activity (distance traveled) in naïve animals measured in OF test

Since differences in basal locomotor activity can interfere with measures in other behavioral tests, including the EPM, OF data reported in the included studies were meta-analyzed for parameters used to assess locomotor activity. The pooled results of six studies showed no significant influence of chronic fluoxetine on locomotor activity compared to control in naïve animals (SMD =  – 0.14; *p* = 0.63; test for heterogeneity: Chi^2^ = 25.38; df = 7; *p* = 0.0007; I^2^ = 72%) (Fig. 4), and the effect remained unchanged in leave-one-out analyses.

#### Stress-exposed animals

**Fig. 5:**
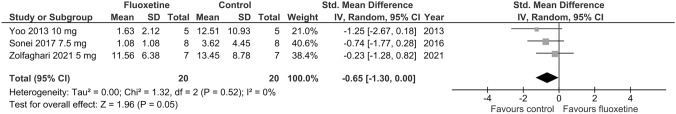
Forest plot of the effects of chronic fluoxetine at doses of 5–10 mg/kg/day vs. vehicle on anxiety-like behavior in stress-exposed animals measured as time spent in the open arms of the EPM

Three trials assessed anxiety-like behavior using a measure of time spent in the open arms of the EPM in stress-exposed rodents. Two studies used male rats [63, 68] while one study was conducted on a female population [57]. Stress-inducing procedures included maternal separation during the first two weeks of life [57, 68] or 4-week adolescent stress exposure induced by social isolation [63]. Fluoxetine doses ranged from 5 to 10 mg/kg/day. As shown in Fig. 5, there were not significant differences between chronic adolescent fluoxetine and vehicle in the time spent in the open arms of the EPM (SMD =  – 0.65; *p* = 0.05; test for heterogeneity: Chi^2^ = 1.32; df = 2; *p* = 0.52; *I*^2^ = 0%). Sensitivity study analyses showed significance (*p* = 0.03) only after the exclusion of Zolfaghari results [68] where a lower—5 mg/kg/day fluoxetine dose was used. However, conclusions from the analysis of anxiety-like behavior in stress-exposed animals were strongly limited since it was based on 3 studies only that prevented any subgroup analyses. No heterogeneity (0%) was detected across the studies included in the meta-analysis conducted in stress-exposed animals.

**Fig. 6:**
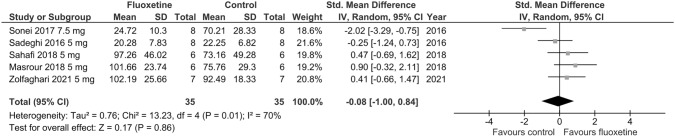
Forest plot of the effects of chronic fluoxetine at doses of 5–7.5 mg/kg/day vs. vehicle on locomotor activity (number of squares crossed) in stress-exposed animals measured in OF test

Based on data from OF test, no significant influence of chronic adolescent fluoxetine on locomotor activity compared to control in stress-exposed animals was reported (SMD =  – 0.08; *p* = 0.86; test for heterogeneity: Chi^2^ = 13.23; df = 4; *p* = 0.01; *I*^2^ = 70%) (Fig. 6), and the effect remained unchanged in leave-one-out analyses.

### Depressive-like behavior

#### Naïve animals

**Fig. 7:**
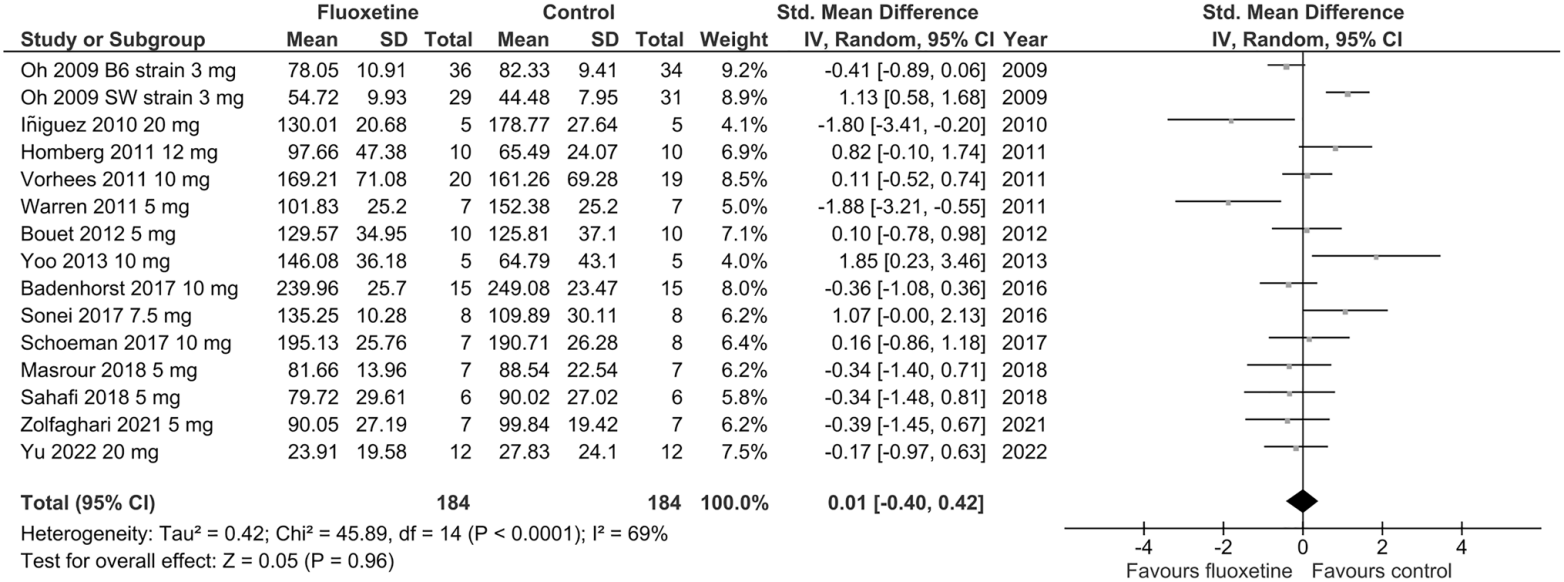
Forest plot of the effects of chronic fluoxetine at doses of 3–20 mg/kg/day vs. vehicle on immobility time in the FST in naïve animals

Fourteen trials assessed the effect of fluoxetine on depressive-like behavior using a measure of immobility time in the FST in naïve rodents. Separated results for two different mouse strains [51] and three different doses of fluoxetine [54] were reported in one study each. Based on pooled data from the included studies, no significant differences between fluoxetine and vehicle were observed in the main meta-analysis (SMD = 0.01; *p* = 0.96; test for heterogeneity: Chi^2^ = 45.89; df = 14; *p* < 0.0001; *I*^2^ = 69%) (Fig. 7) or in subgroup analyses when higher (7.5–20 mg/kg/day) (SMD = 0.19; *p* = 0.48; test for heterogeneity: Chi^2^ = 17.34; df = 7; *p* = 0.02; *I*^2^ = 60%) or lower (3–5 mg/kg/day) (SMD =  – 0.31; *p* = 0.28; test for heterogeneity: Chi^2^ = 34.18; df = 8; *p* < 0.0001; *I*^2^ = 77%) fluoxetine doses were tested (Online Resource ESM_6). Leave-one-out analyses showed no marked difference in the main meta-analysis and subgroup analysis of higher fluoxetine doses (*p* > 0.05). However, the results reached statistical significance (*p* = 0.02) after the exclusion of Swiss Webster mice from the meta-analysis regarding doses of 3–5 mg/kg/day (Online Resource ESM_4), suggesting an antidepressant effect of fluoxetine administered at lower doses. Since only one trial reported results for females [57], and only one trial included mice [51], it was not possible to analyze sex or species effects. There was moderate to high heterogeneity (*I*^2^ > 50%) across the studies included in particular meta-analyses regarding immobility time in the FST.

#### Stress-exposed animals

**Fig. 8:**
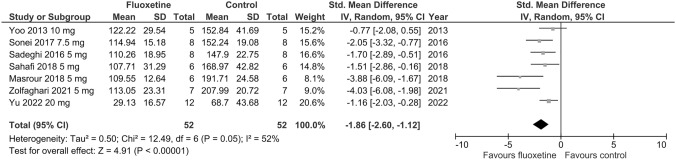
Forest plot of the effects of chronic fluoxetine at doses of 5–10 mg/kg/day vs. vehicle on immobility time in the FST in stress-exposed animals

Seven trials assessed depressive-like behavior using a measure of immobility time in the FST in stress-exposed animals. As shown in Fig. 8, chronic fluoxetine exposure during adolescence significantly decreased immobility time in the FST compared to vehicle (SMD =  – 1.86; *p* < 0.00001; test for heterogeneity: Chi^2^ = 12.49; df = 6; *p* = 0.05; *I*^2^ = 52%). Leave-one-out analyses showed no marked difference in the meta-analysis results (*p* < 0.0001). Limited number of studies included in the comparison prevented any subgroup analyses. High heterogeneity (*I*^2^ > 50%) was detected across the studies included in the meta-analysis regarding immobility time in the FST in stress-exposed animals.

## Discussion

To the best of our knowledge, this study is the first comprehensive review regarding the effects of chronic fluoxetine exposure on anxiety- and depressive-like behavioral measures during adolescence in animal studies. The results of our meta-analysis suggest that in naïve animals anxiogenic-like effects of chronic fluoxetine administration measured as a reduction in time spent in the open arms of the EPM may be dose-related.

The EPM is one of the most frequently used tests for measuring anxiety-like behavior in rodents, and its validation has been performed with several anxiolytic and anxiety-inducing drugs [114]. The performed meta-analysis showed that higher (7.5–20 mg/kg/day), but not lower (3–5 mg/kg/day), doses of chronic fluoxetine during adolescence exerted anxiogenic-like effects. In humans, fluoxetine is indicated at 20–60 mg/day [115], corresponding to approximately 0.3–0.9 mg/kg. In MDD, a fluoxetine dose of 20 mg/day is effective in adolescents [116, 117] and adults [118] and corresponds to an approximately 100 ng/ml fluoxetine plasma level and approximately 200 ng/ml fluoxetine/norfluoxetine. Due to higher hepatic drug metabolism in rodents, fluoxetine, as other drugs is commonly administered at approximately tenfold higher doses than in humans [31]. In animal studies, fluoxetine administration of 3–10 mg/kg/day resulted in serum fluoxetine and norfluoxetine levels within the ranges observed and recommended in adult and adolescent humans under fluoxetine treatment [51, 103, 116, 119–123]. Higher doses (~ 18 mg/kg/day) correspond to very high human plasma levels, which in the case of 25 mg/kg/day are far greater than the normal therapeutic level [51, 121, 122], and the effects of such a high dose can result in a loss of specificity for the serotonergic system [121]. However, there have been broad variations in serum fluoxetine and norfluoxetine levels after similar fluoxetine dosing in animal studies. A comparable fluoxetine dose of approximately 3–4 mg/kg/day in mice resulted in more than two times higher fluoxetine and norfluoxetine serum levels in the latter study [51, 81]. Hodes et al. [124] reported plasma levels of fluoxetine and norfluoxetine after 10 mg/kg/day dosing comparable to those reported for approximately 20 mg/kg/day in Dulawa’s et al. study [121]. Discrepancies in fluoxetine and its metabolite levels after similar fluoxetine dosing in animal studies reported in literature could be explained by species/strain differences in drug metabolism (since even in individual humans, there are significant differences in the metabolism of fluoxetine [117, 119, 125, 126]) or not performing the analysis under steady-state conditions.

The route of administration is also an important factor influencing the pharmacokinetics of the drug, thus affecting the plasma concentration of fluoxetine and its metabolite. In animal studies, fluoxetine is administered mostly intraperitoneally or orally in drinking water. The latter eliminates sharp peaks in drug levels after injection and stress induced by the administration. Furthermore, in rodents, continuous administration of fluoxetine in drinking water corresponds better with the clinical situation since the half-lives of fluoxetine and norfluoxetine are much shorter than those in humans (5–6 h vs. 1–3 days for fluoxetine and 12.3–15 h vs. 7–15 days for norfluoxetine) [127–129]. Unfortunately, in our study, we were not able to analyze results depending on the route of administration since, in very few trials, fluoxetine was administered in drinking water. In conclusion, doses between 3 and 5 mg/kg/day in animal models correspond best with plasma levels of fluoxetine and norfluoxetine reported in adolescent humans treated with a fluoxetine dose of 20 mg/day; however, doses up to 10 mg/kg/day lead to serum levels of fluoxetine and its metabolite within the ranges reported for therapeutic dosages in humans.

Among the included studies on naïve animals, only one trial reported significant anxiogenic effects of a lower (3 mg/kg/day) dose of fluoxetine in the EPM (in C57Bl/6 mice but not in Swiss Webster mice) [51]. However, it was the only study included in the meta-analysis in which fluoxetine administration was initiated in juveniles (PD 14). Since the 5-HT system is highly engaged in anxiety behavior, and it matures during early postnatal development [130–133], early-life fluoxetine-induced modulation of the 5-HT neurotransmission should be considered when discussing the anxiogenic effects of low fluoxetine doses in this study. Moreover, anxiogenic effects of chronic fluoxetine in C57Bl/6 mice, but not BALB/c mice, were also shown in adult animals [134], suggesting that genetic background could be responsible for specific effects of fluoxetine treatment in C57Bl/6 mice.

In naïve animals, pooled results showed anxiogenic-like effects of chronic adolescent fluoxetine in males but not in females. It should be mentioned that the comparison in female population was based on 3 studies only, thus the conclusions are strongly limited. However, sex discrepancies in analyzed outcome need more discussion. In untreated, late-adolescent rats, females were characterized by significantly increased locomotor activity and longer time spent in the open arms of the EPM compared to males [135]. Moreover, increased locomotor activity after chronic fluoxetine, which is a confounding factor in the context of the EPM test [136], was observed in female but not in male adolescent rats [67]. The above observations suggest that sex-dependent differences in modulation of anxiaty-like behaviors by chronic adolescent fluoxetine might be confounded by sex differences in exploratory behavior. However, clear sex-dependent effects of adolescent fluoxetine on anxiety-like behavior without corresponding changes in locomotor activity were reported in early adulthood (PD 90). Specifically, chronic adolescent drug administration decreased the time spent in the open arms of the EPM in males but not in females. The increase in anxiety was also dose-dependent since only a higher (10 mg/kg/day), but not a lower (5 mg/kg/day), dose of fluoxetine reduced the time spent in the open arms of the EPM in males [78].

It should be emphasized that our results are in line with studies conducted in adult animals, in which chronic fluoxetine produced anxiogenic-like [137–139], rather than anxiolytic-like effects in the EPM [140]. Among the few behavioral tests that demonstrate anxiolytic-like effects of chronic antidepressant treatment in adult rodents are novelty-suppressed feeding (NSF) and novelty-induced hypophagia (NIH), which refer to the inhibition of feeding in response to a novel (and thus anxiogenic) environment. In these cases, the anxiolytic activity of chronic fluoxetine was reported both in adult rats and mice across a broad spectrum of doses [122, 141–145]. Three of the studies included in our systematic review regarding adolescents also assessed latency to feed in the novel environment to measure anxiety-like behavior [51, 52, 69]. Interestingly, a significant increase in anxiogenic-like behavior was demonstrated in two primary studies in naïve adolescents regardless of fluoxetine dose (3 and 20 mg/kg) and species (rat and two mouse strains) [51, 52]. Moreover, Oh et al. reported age-dependent effects of chronic fluoxetine in the NIH test, with anxiogenic-like behavior in adolescents and anxiolytic-like effects in adult mice [51]. Thus, although the anxiety measures might differ depending on the behavioral test used, the age factor seems to be crucial.

Discussing behavioral manifestations of anxiety-like effects of adolescent fluoxetine treatment, long-lasting effects, reported in early adulthood (PD 70–90), should be mentioned. In naive animals, a few weeks after drug cessation, anxiety measures in the EPM were increased [52, 55, 73, 75, 78] (in the case of [78] only in males at a dose of 10 mg/kg/day) or unchanged [51, 77, 78] (in males and females at a dose of 5 mg/kg/day [78] and in females at a dose of 10 mg/kg/day [71] or only in wild-type animals [79]). However, in a genetic mouse model of increased anxiety, chronic fluoxetine in adolescence increased the time spent in the open arms of the EPM and reduced the latency to feed in the NIH test in early adulthood, reflecting the anxiolytic effects of the drug in this phenotype of mice [71]. It would be interesting to relate the results obtained in naïve rodents to that reported in animal models of depression.

Although our meta-analysis showed that adolescent fluoxetine administration did not significantly affect anxiety level in stress-exposed animals, the comparison was based on very few data, making it difficult to draw the conclusions.

In naïve animals, the pooled results of the included studies suggest a lack of chronic adolescent fluoxetine effects on depressive-like behaviors, measured as immobility time in the FST. No dose-dependent effects were shown. In the FST, depressive-like behavior is measured as an increased duration of immobility, reflecting the level of behavioral despair or rather an adaptation and switch from active to passive coping strategy. As a decrease in immobility in the FST, as well as time spent in the open arms of the EPM, can be confused with a drug-induced increase in locomotor activity, a meta-analysis of results regarding total distance traveled in the OF test was performed, showing that chronic fluoxetine did not induce hyperactivity. The interpretation of the obtained results is, however, complicated since the FST’s validity is still debated [146]. The main limitation of the FST is that acute antidepressant administration immediately decreases the duration of immobility, while the clinical onset of conventional therapies is observed after weeks [147]. Antidepressant-like effects of acute fluoxetine in adolescent animals have been reported [52, 148, 149]. However, in the two mentioned studies, statistically significant effects were reported when swimming behaviors were measured, while there was only a trend toward a drug-induced reduction in immobility time [52, 149]. Therefore, the meta-analysis of other, less often reported behavioral measures of the FST would give a more complex picture of the depressive-like effects of chronic adolescent fluoxetine in naïve animals.

On the contrary, our meta-analysis showed a sensitivity of the FST on measures of antidepressant-like effects of chronic adolescent fluoxetine in animals with stress exposure. Chronic adolescent fluoxetine significantly decreased immobility time in the FST compared to controls. Above results suggest that chronic fluoxetine administration in adolescence reverses depressive-like behavior induced by chronic stress exposure. Interestingly, fluoxetine antidepressant-like effects were consistent in meta-analyzed studies regardless of the stress paradigm used. In most trials animals were subjected to neonatal maternal separation with subsequent fluoxetine treatment [57, 60, 64, 65, 68]. However, in some studies stress procedure and fluoxetine administration were simultaneous [63, 69] and based on social isolation or chronic unpredictable mild stress paradigm. It suggests that fluoxetine can reverse depressive-like behavior or prevent their emergence during stress exposure.

Among the multiple mechanisms underlying the observed anxiogenic-like effects of chronic fluoxetine in adolescence, changes in the serotoninergic system would be pivotal since fluoxetine primarily acts on the 5-HT system. In adult animals, a persistent increase in 5-HT levels in several brain regions after chronic fluoxetine exposure was reported [150–154]. In adolescent animals, ten days of fluoxetine treatment at a dose of 10 mg/kg/day resulted in reduced serotonin levels in the prefrontal cortex and raphe nucleus or no change in the hippocampus, while only hypothalamic 5-HT levels were increased [57, 59]. It could be proposed that, after excessive stimulation of the serotoninergic system by high doses of fluoxetine in animals with normal basal levels of 5-HT, compensatory changes in the developing brain emerge, leading to hypoactivity of the 5-HT system. Low 5-HT activity was related to hypersensitivity to mild stressors and increased anxiety [155] and impulsive and aggressive behaviors and was suggested to be responsible for impulsivity and suicidal ideation during the first weeks of fluoxetine treatment in humans [156]. Interestingly, as recently shown, adolescent modulation of the 5-HT system through blockade of 5-HT-1A receptors resulted in increased anxiety without impacting depression-like behaviors in adulthood, suggesting that this developmental period is sensitive to specific changes in 5-HT signaling through the 5-HT-1A receptor [35]. Chronic SSRI treatment is known to produce not only desensitization of 5-HT-1A autoreceptors but also changes in postsynaptic 5-HT-1A serotonin receptor function [157, 158] and involvement of 5-HT-1A receptors localized in the paraventricular nucleus of the hypothalamus (PVN) in anxiety and depression is suggested [35, 159]. Thus, chronic administration of SSRIs during the developmental stage, when the plasticity of the 5-HT system is observed, could lead to a modulation of anxiety- and depressive-like behaviors.

The main limitation of our study is the relatively small number of studies suitable for aggregation, which prevented the pre-planned subgroup analysis and strongly limited conclusions regarding fluoxetine administration on anxiety-like behavior in stress-exposed animals. Most of the included studies were performed on males; therefore, meta-analyses conducted in the female population were restricted to anxiety-like behavior only and based on limited data. It is an important limitation since clinical data suggest that women are more commonly diagnosed with mood disorders over their lifetime [160] and that the prevalence of depressive and anxiety disorders in females compared to males is greatest during adolescence [6]. Moreover, factors such as species/strain, sex, dose, schedule of fluoxetine treatment (treatment duration, route of fluoxetine administration or drug-free intervals) and day of behavioral assessment contributed to the heterogeneity of the studies. Another limitation is that our findings should be considered as relating mostly to Wistar rats, while as reported previously, the antidepressant-like effects of fluoxetine in the FST might be strain-dependent [121, 161].

Furthermore, when interpreting the results of behavioral tests, many confounding factors should be considered. First, using more than one test over a brief period of time could influence the results of the proceeding behavioral tests [162], and in most of the included studies, animals were assessed in a series of tests. Furthermore, experimental conditions appear to be crucial for modifying the overall outcomes of anxiety-related behaviors in rodents. According to the literature regarding the EPM, handling of the animals [163] and illumination [164] and even time of the day/night cycle could contribute to the results, thus generating heterogeneity of results. On the other hand, a meta-analysis of heterologous studies could strengthen the obtained results, proving that drug factors are crucial. Interpretation of individual studies included in the meta-analysis shows the need to develop tests for complex measures of emotional reactivity since individual tests assess only a part of complex animals’ emotional profiles. These limitations could be overcome using a battery of tests; however, such an approach requires intertest intervals, and previous test experience could influence the results. There is also a need for a good animal model valid for the assessment of chronic adolescent drug exposure that mirrors the delayed response to antidepressants in the clinic. Future preclinical studies with direct comparisons between males and females and between species/strains are needed. Ideally, an assessment of the brain and plasma levels of fluoxetine and norfluoxetine should be performed during treatment to compare metabolic and neuronal responses between studies and clinical conditions.

While three of the included primary studies [54, 59, 62] reported results for more than one dose of fluoxetine and compared it with the same control group, for the meta-analysis a selection of one dose from every single study has been done (otherwise animals from control groups would be included twice in the same comparison). Although in the main meta-analysis the results reported for the middle value of the dose range (10 mg/kg/day)  were included, this should be considered as another limitation of our study.

Among other limitations of our review language bias should be mentioned as the search strategy was limited to English language publications. What is more, the implementation of grey literature in our study could reduce the publication bias; however, some authors suggest that non-peer-reviewed studies may induce another bias if include data that failed to be published due to its lower quality [165].

The assessment of the risk of bias in the studies included in the meta-analysis was performed only by one reviewer, while it would be more correct to perform it independently by the two reviewers. However, many animal studies do not adequately report information necessary for the assessment of study quality. Lack of information about randomization method used and blinding of the investigators and outcome assessors in most of the included primary studies is another factor that biased the obtained results.

In summary, our results confirmed that in animal models of depression adolescent fluoxetine reduced depressive-like behaviors without an increase in anxiety. However, in naïve animals, adolescent fluoxetine administration increased anxiety-like behavior, suggesting an influence on properly maturing rodent brain. Higher doses of the drug, partially corresponding to the range reported for therapeutic dosages in humans and commonly used in animal studies, induced anxiogenic-like effects. However, when the drug was administered in lower doses, best reflecting the dose of fluoxetine most commonly used in adolescent humans, the anxiety-like behavior was unchanged. Nonetheless, in naïve animals depressive-like behaviors were not influenced by chronic adolescent fluoxetine administration regardless of the dose analyzed.

Anxiety has been reported among adult and adolescent humans undergoing fluoxetine therapy [166–168]. Beasley et al. [125] showed that anxiety was significantly increased only in patients under treatment with higher doses of fluoxetine (60 mg/day). Additionally, in the first report of a potential increase in suicidality due to fluoxetine treatment, four of the six patients who developed suicidality under therapy received high doses of the drug (60–80 mg/day), and some of them also reported an increase in anxiety during treatment [169]. Although clinical implications of fluoxetine exposure cannot be drawn directly from animal studies, the current results raise further concerns about the use of high doses of fluoxetine. Moreover, because adolescence is a critical period of 5-HT system plasticity/maturation and the emergence of anxiety and mood disorders in humans, adolescent patients under fluoxetine treatment should be carefully monitored.

## Supplementary Information

Below is the link to the electronic supplementary material.

Supplementary file 1 Search strategy construction for the Medline (via PubMed), Web of Science Core Collection and ScienceDirect (last updated: 12.04.2022) (PDF 153 KB)

Supplementary file 2 Raw data extracted from the primary studies (PDF 142 KB)

Supplementary file 3 Funnel plots addressing publication bias of studies included in comparison that evaluated: time spent in the open arms of the EPM in naïve animals (A), locomotor activity in the OF in naïve animals (B), immobility time in the FST in naïve animals (C) (PDF 536 KB)

Supplementary file 4 Sensitivity analysis results (PDF 285 KB)

Supplementary file 5 Forest plot of effects of chronic fluoxetine at higher (7.5-20 mg/kg/day) or lower (3-5 mg/kg/day) doses vs. vehicle on anxiety-like behavior in naïve animals measured as time spent in the open arms of the EPM. Note: Significance of the results was unchanged when effects of fluoxetine in the dose of 20 mg instead of 10 mg from Amodeo’s et al. study were included in the higher dose subgroup (Online Resource ESM_4) (PDF 902 KB)

Supplementary file 6 Forest plot of the effects of chronic fluoxetine at higher (7.5-20 mg/kg/day) or lower (3-5 mg/kg/day) doses vs. vehicle on despair-like behavior in naïve animals measured as immobility time in the FST (PDF 1317 KB)

## Data Availability

All data analyzed during this study are included in this published article (Online Resource ESM_2).

## Abbreviations

**CI**: Confidence intervals
**CPP**: Cocaine place preference
**EPM**: Elevated plus maze
**ESM**: Electronic supplementary materials
**FDA**: Food and drug administration
**FST**: Forced swim test
**MDD**: Major depressive disorder
**MDE**: Major depressive episode
**MeSH**: Medical subject heading
**NIH**: Novelty-induced hypophagia
**NSF**: Novelty-suppressed feeding
**OCD**: Obsessive-compulsive disorder
**OF**: Open field
**PD**: Postnatal day
**PICO**: Population, intervention, comparison and outcome
**PVN**: Paraventricular nucleus of the hypothalamus
**SMD**: Standardized mean difference
**SSRIs**: Selective serotonin reuptake inhibitors
