# Supplementary material for: Effects of chronic fluoxetine treatment on anxiety- and depressive-like behaviors in adolescent rodents – systematic review and meta-analysis
Source: Pharmacol Rep. 2022 Sep 24;74(5):920–46. doi: 10.1007/s43440-022-00420-w (PMC9584991; doi:10.1007/s43440-022-00420-w)
Supplement: Supplementary file 2 — Raw data extracted from the primary studies (PDF 142 KB) [file 43440_2022_420_MOESM2_ESM.pdf]

Raw data extracted from the primary studies

	Data used for comparison of the effects of chronic fluoxetine at doses of 3-20 mg/kg/day vs vehicle on anxiety-like behavior measured as time spent in the open arms of the EPM							
		Fluoxetine			Control			Comments
**Study/population**	**Fluoxetine dose**	**Mean**	**SD**	**N**	**Mean**	**SD**	**N**	
Oh 2009 [51] B6 strain	3 mg	4.22	1.11	36	11.60	2.34	34	
Oh 2009 [51] SW strain	3 mg	6.08	1.97	29	6.53	2.44	31	
Iñiguez 2010 [52]	20 mg	5.62	3.34	8	22.07	13.10	8	
Homberg 2011 [53]	12 mg	13.84	8.87	10	23.78	9.28	10	data obtained from the Authors of primary study
Vorhees 2011 [54]	10 mg	21.58	15.99	20	25.45	15.72	20	
Warren 2011 [55]	5 mg	30.77	9.30	7	34.36	12.34	7	
Yoo 2013 [57]	10 mg	5.56	5.17	5	14.78	11.63	5	
Amodeo 2015 [59] Female	10 mg	41.34	13.10	9	46.77	10.55	9	
Amodeo 2015 [59] Male	10 mg	39.52	14.38	9	43.15	8.31	9	
Sonei 2017 [63]	7.5 mg	5.37	4.29	8	12.90	3.24	8	
Sadegzadeh 2020 [67] Female	5 mg	49.17	17.89	8	29.91	17.89	8	
Sadegzadeh 2020 [67] Male	5 mg	40.61	24.96	8	38.47	17.14	8	
Zolfaghari 2021 [68]	5 mg	14.25	5.19	7	36.46	6.79	7	
	**Data used for comparison of the effects of chronic fluoxetine at higher (7.5-20 mg/kg/day) or lower (3-5 mg/kg/day) doses vs vehicle on anxiety-like behavior measured as time spent in the open arms of the EPM**							
**Higher doses**								
Iñiguez 2010 [52]	20 mg	5.62	3.34	8	22.07	13.10	8	
Homberg 2011 [53]	12 mg	13.84	8.87	10	23.78	9.28	10	data obtained from the Authors of primary study
Vorhees 2011 [54]	10 mg	21.58	15.99	20	25.45	15.72	20	
Yoo 2013 [57]	10 mg	5.56	5.17	5	14.78	11.63	5	
Amodeo 2015 [59] Female	10 mg	41.34	13.10	9	46.77	10.55	9	
Amodeo 2015 [59] Male	10 mg	39.52	14.38	9	43.15	8.31	9	
Sonei 2017 [63]	7.5 mg	5.37	4.29	8	12.90	3.24	8	
**Lower doses**								

Oh 2009 [51] B6 strain	3 mg	4.22	1.11	36	11.60	2.34	34	
Oh 2009 [51] SW strain	3 mg	6.08	1.97	29	6.53	2.44	31	
Vorhees 2011 [54]	3 mg	22	15.99	20	25.45	15.72	20	
Warren 2011 [55]	5 mg	30.77	9.30	7	34.36	12.34	7	
Amodeo 2015 [59] Female	5 mg	48.58	13.74	9	46.77	10.55	9	
Amodeo 2015 [59] Male	5 mg	44.42	13.10	9	43.15	8.31	9	
Sadegzadeh 2020 [67] Female	5 mg	49.17	17.89	8	29.91	17.89	8	
Sadegzadeh 2020 [67] Male	5 mg	40.61	24.96	8	38.47	17.14	8	
Zolfaghari 2021 [68]	5 mg	14.25	5.19	7	36.46	6.79	7	
	**Data used for comparison of the effects of chronic fluoxetine at doses of 3-20 mg/kg/day vs vehicle on anxiety-like behavior in male measured as time spent in the open arms of the EPM**							
Oh 2009 [51] B6 strain	3 mg	4.22	1.11	36	11.60	2.34	34	
Oh 2009 [51] SW strain	3 mg	6.08	1.97	29	6.53	2.44	31	
Iñiguez 2010 [52]	20 mg	5.62	3.34	8	22.07	13.10	8	
Vorhees 2011 [54]	10 mg	21.58	15.99	20	25.45	15.72	20	
Warren 2011 [55]	5 mg	30.77	9.30	7	34.36	12.34	7	
Homberg 2011 [53]	12 mg	13.84	8.87	10	23.78	9.28	10	data obtained from the Authors of primary study
Amodeo 2015 [59] Male	10 mg	39.52	14.38	9	43.15	8.31	9	
Sonei 2017 [63]	7.5 mg	5.37	4.29	8	12.90	3.24	8	
Sadegzadeh 2020 [67] Male	5 mg	40.61	24.96	8	38.47	17.14	8	
Zolfaghari 2021 [68]	5 mg	14.25	5.19	7	36.46	6.79	7	
	**Data used for comparison of the effects of chronic fluoxetine at doses of 3-20 mg/kg/day vs vehicle on locomotor activity measured in OF test**							
Oh 2009 [51] B6 strain	3 mg	2656	495	36	3102	506	34	
Oh 2009 [51] SW strain	3 mg	4110	970	18	4011	791	18	
Iñiguez 2010 [52]	20 mg	12210	4990	10	13780	5470	10	
Homberg 2011 [53]	12 mg	4866	1666	10	4617	1412	10	
Sass 2012 [56]	10 mg	3591	1063	12	4926	750	12	
Yoo 2013 [57]	10 mg	1977	806	5	2265	391	5	
Sadegzadeh 2020 [67] Female	5 mg	2392	653	8	2015	609	8	

Sadegzadeh 2020 [67] Male	5 mg	3208	587	8	2423	435	8	
	**Data used for comparison of the effects of chronic fluoxetine at doses of 3-20 mg/kg/day vs vehicle on depressive-like behavior measured as immobility time in the FST**							
Oh 2009 [51] B6 strain	3 mg	78.05	10.91	36	82.33	9.41	34	
Oh 2009 [51] SW strain	3 mg	54.72	9.93	29	44.48	7.95	31	
Iñiguez 2010 [52]	20 mg	130.01	20.68	5	178.77	27.64	5	
Homberg 2011 [53]	12 mg	97.66	47.38	10	65.49	24.07	10	
Vorhees 2011 [54]	10 mg	169.21	71.08	20	161.26	69.28	19	
Warren 2011 [55]	5 mg	101.83	25.20	7	152.38	25.20	7	
Bouet 2012 [32]	5 mg	129.57	34.95	10	125.81	37.10	10	
Yoo 2013 [57]	10 mg	146.08	36.18	5	64.79	43.10	5	
Badenhorst 2017 [61]	10 mg	239.96	25.70	15	249.08	23.47	15	
Sonei 2017 [63]	7.5 mg	135.25	10.28	8	109.89	30.11	8	
Schoeman 2017 [62]	10 mg	195.13	25.76	7	190.71	26.28	8	
Masrour 2018 [65]	5 mg	81.66	13.96	7	88.54	22.54	7	
Sahafi 2018 [64]	5 mg	79.72	29.61	6	90.02	27.02	6	
Zolfaghari 2021 [68]	5 mg	90.05	27.19	7	99.84	19.42	7	
Yu 2022 [69]	20 mg	23,91	19,58	12	27,83	24,10	12	
	**Data used for comparison of the effects of chronic fluoxetine at higher (7.5-20 mg/kg/day) or lower (3-5 mg/kg/day) doses vs vehicle on depressive-like behavior measured as immobility time in the FST**							
**Higher doses**								
Iñiguez 2010 [52]	20 mg	130.01	20.68	5	178.77	27.64	5	
Homberg 2011 [53]	12 mg	97.66	47.38	10	65.49	24.07	10	
Vorhees 2011 [54]	10 mg	169.21	71.08	20	161.26	69.28	19	
Yoo 2013 [57]	10 mg	146.08	36.18	5	64.79	43.10	5	
Badenhorst 2017 [61]	10 mg	239.96	25.70	15	249.08	23.47	15	
Sonei 2017 [63]	7.5 mg	135.25	10.28	8	109.89	30.11	8	
Schoeman 2017 [62]	10 mg	195.13	25.76	7	190.71	26.28	8	
Yu 2022 [69]	20 mg	23,91	19,58	12	27,83	24,10	12	
**Lower doses**								
Oh 2009 [51] B6 strain	3 mg	78.05	10.91	36	82.33	9.41	34	
Oh 2009 [51] SW strain	3 mg	54.72	9.93	29	44.48	7.95	31	
Warren 2011 [55]	5 mg	101.83	25.20	7	152.38	25.20	7	

Vorhees 2011 [54]	3 mg	154.64	69.80	21	161.26	69.28	19	
Bouet 2012 [32]	5 mg	129.57	34.95	10	125.81	37.10	10	
Schoeman 2017 [62]	5 mg	148.23	25.76	7	190.71	26.28	8	
Masrour 2018 [65]	5 mg	81.66	13.96	7	88.54	22.54	7	
Sahafi 2018 [64]	5 mg	79.72	29.61	6	90.02	27.02	6	
Zolfaghari 2021 [68]	5 mg	90.05	27.19	7	99.84	19.42	7	
	**Data used for comparison of the effects of chronic fluoxetine at doses of 3-20 mg/kg/day vs vehicle on anxiety-like behavior measured as time spent in the open arms of the EPM in stress-exposed animals**							
Yoo 2013 [57]	10 mg	1.63	2.12	5	12.51	10.93	5	
Sonei 2017 [63]	7.5 mg	1.08	1.08	8	3.62	4.45	8	
Zolfaghari 2021 [68]	5 mg	11.56	6.38	7	13.45	8.78	7	
	**Data used for comparison of the effects of chronic fluoxetine at doses of 3-20 mg/kg/day vs vehicle on horizontal activity (number of squares crossed) measured in OF test in stress-exposed animals**							
Sedeghi 2016 [60]	5 mg	20.28	7.83	8	22.25	6.82	8	
Sonei 2017 [63]	7.5 mg	24.72	10.30	8	70.21	28.33	8	
Masrour 2018 [65]	5 mg	101.66	23.74	6	75.76	29.30	6	
Sahafi 2018 [64]	5 mg	97.26	46.02	6	73.16	49.28	6	
Zolfaghari 2021 [68]	5 mg	102.19	25.66	7	92.49	18.33	7	
	**Data used for comparison of the effects of chronic fluoxetine at doses of 3-20 mg/kg/day vs vehicle on depressive-like behavior measured as immobility time in the FST in stress-exposed animals**							
Yoo 2013 [57]	10 mg	122.22	29.54	5	152.84	41.69	5	
Sedeghi 2016 [60]	5 mg	110.26	18.95	8	147.90	22.75	8	
Sonei 2017 [63]	7.5 mg	114.94	15.18	8	152.24	19.08	8	
Masrour 2018 [65]	5 mg	109.55	12.64	6	191.71	24.58	6	
Sahafi 2018 [64]	5 mg	107.71	31.29	6	168.97	42.82	6	
Zolfaghari 2021 [68]	5 mg	113.05	23.31	7	207.99	20.72	7	
Yu 2022 [69]	20 mg	29,13	16,57	12	68,70	43,68	12	
