# Supplementary material for: Effects of chronic fluoxetine treatment on anxiety- and depressive-like behaviors in adolescent rodents – systematic review and meta-analysis
Source: Pharmacol Rep. 2022 Sep 24;74(5):920–46. doi: 10.1007/s43440-022-00420-w (PMC9584991; doi:10.1007/s43440-022-00420-w)
Supplement: Supplementary file 3 — Funnel plots addressing publication bias of studies included in comparison that evaluated: time spent in the open arms of the EPM in naïve animals (A), locomotor activity in the OF in naïve animals (B), immobility time in the FST in naïve animals (C) (PDF 536 KB) [file 43440_2022_420_MOESM3_ESM.pdf]

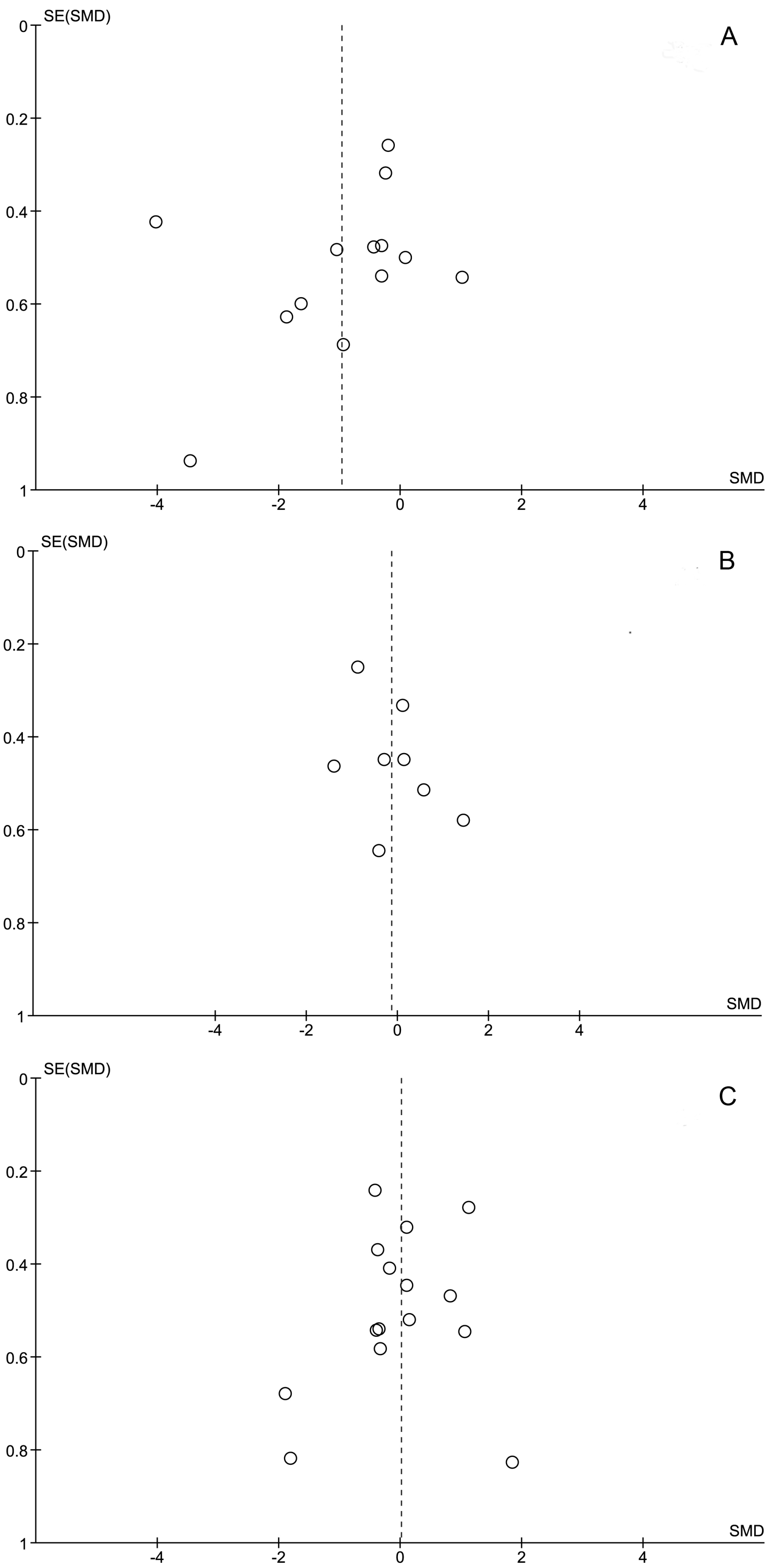

Funnel plots addressing publication bias of studies included in comparison that evaluated: time spent in the open arms of the EPM in naive animals (A), locomotor activity in the OF in naive animals (B), immobility time in the FST in naive animals (C).
