# Supplementary material for: Effects of chronic fluoxetine treatment on anxiety- and depressive-like behaviors in adolescent rodents – systematic review and meta-analysis
Source: Pharmacol Rep. 2022 Sep 24;74(5):920–46. doi: 10.1007/s43440-022-00420-w (PMC9584991; doi:10.1007/s43440-022-00420-w)
Supplement: Supplementary file 4 — Sensitivity analysis results (PDF 285 KB) [file 43440_2022_420_MOESM4_ESM.pdf]

Sensitivity analysis results

Sensitivity analysis regarding effects of chronic fluoxetine at doses of 3-20 mg/kg/day vs vehicle on anxiety-like behavior measured as time spent in the open arms of the EPM – leave one-out study analyses		
	SMD [95% CI]; p	Test for heterogeneity
Excluding Homberg 2011 study [53]	SMD = -0.96 [95% CI: -1.76; -0.17]; Z = 2.38; p = 0.02	Tau^2 = 1.67; Chi^2 = 95.93; df = 11; p< 0.00001; I^2 = 89%
Excluding Iniguez 2010 study [52]	SMD = -0.92 [95% CI: -1.69; -0.14]; Z = 2.32; p = 0.02	Tau^2 = 1.58; Chi^2 = 94.13; df = 11; p< 0.00001; I^2 = 88%
Excluding Sonei 2017 study [63]	SMD = -0.90 [95% CI: -1.66; -0.13]; Z = 2.29; p = 0.02	Tau^2 = 1.56; Chi^2 = 93.05; df = 11; p< 0.00001; I^2 = 88%
Excluding Zolfaghari 2021 study [68]	SMD = -0.81 [95% CI: -1.55; -0.08]; Z = 2.18; p = 0.03	Tau^2 = 1.42; Chi^2 = 87.94; df = 11; p< 0.00001; I^2 = 87%
Analysis regarding effects of chronic fluoxetine at higher (7.5-20 mg/kg/day) doses vs vehicle on anxiety-like behavior measured as time spent in the open arms of the EPM – inclusion of results for dose of 20 mg instead of 10 mg from Amodeo 2015 study		
Amodeo 2015 study [59] – dose 20 mg	SMD = -1.24 [95% CI: -1.85; -0.64]; Z = 4.03; p < 0.0001	Tau2 = 0.36; Chi2 = 13.99; df = 6; p= 0.03; I2 = 57%

Sensitivity analysis regarding effects of chronic fluoxetine at lower (3-5 mg/kg/day) doses vs vehicle on despair-like behaviour measured as immobility time in FST - leave-one-out study analyses		
Excluding SW strain from Oh 2009 study [51]	SMD = -0.46 [95% CI: -0.84; -0.08]; Z = 2.37; p = 0.02	$Tau^2 = 0.09$; $Chi^2 = 10.39$; df = 7; p = 0.17; $I^2 = 33\%$
