# Supplementary material for: Effects of chronic fluoxetine treatment on anxiety- and depressive-like behaviors in adolescent rodents – systematic review and meta-analysis
Source: Pharmacol Rep. 2022 Sep 24;74(5):920–46. doi: 10.1007/s43440-022-00420-w (PMC9584991; doi:10.1007/s43440-022-00420-w)
Supplement: Supplementary file 5 — Forest plot of effects of chronic fluoxetine at higher (7.5-20 mg/kg/day) or lower (3-5 mg/kg/day) doses vs. vehicle on anxiety-like behavior in naïve animals measured as time spent in the open arms of the EPM. Note: Significance of the results was unchanged when effects of fluoxetine in the dose of 20 mg instead of 10 mg from Amodeo’s et al. study were included in the higher dose subgroup (Online Resource ESM_4) (PDF 902 KB) [file 43440_2022_420_MOESM5_ESM.pdf]

Study or Subgroup	Fluoxetine Mean	Fluoxetine SD	Fluoxetine Total	Control Mean	Control SD	Control Total	Weight	Std. Mean Difference IV, Random, 95% CI	Year
**1.2.1 Higher doses**									
Iñiguez 2010 20 mg	5.62	3.34	8	22.07	13.1	8	5.9%	-1.63 [-2.80, -0.45]	2010
Homberg 2011 12 mg	13.84	8.87	10	23.78	9.28	10	6.4%	-1.05 [-2.00, -0.10]	2011
Vorhees 2011 10 mg	21.58	15.99	20	25.45	15.72	20	7.0%	-0.24 [-0.86, 0.38]	2011
Yoo 2013 10 mg	5.56	5.17	5	14.78	11.63	5	5.5%	-0.93 [-2.27, 0.42]	2013
Amodeo 2015 Female 10 mg	41.34	13.1	9	46.77	10.55	9	6.4%	-0.43 [-1.37, 0.50]	2015
Amodeo 2015 Male 10 mg	39.52	14.38	9	43.15	8.31	9	6.4%	-0.29 [-1.22, 0.64]	2015
Sonei 2017 7.5 mg	5.37	4.29	8	12.9	3.24	8	5.7%	-1.87 [-3.11, -0.64]	2016
**Subtotal (95% CI)**			**69**			**69**	**43.3%**	**-0.79 [-1.26, -0.32]**	
Heterogeneity: Tau² = 0.15; Chi² = 9.64, df = 6 (P = 0.14); I² = 38%									
Test for overall effect: Z = 3.31 (P = 0.0009)									
**1.2.2 Lower doses**									
Oh 2009 B6 strain 3 mg	4.22	1.11	36	11.6	2.34	34	6.6%	-4.02 [-4.85, -3.19]	2009
Oh 2009 SW strain 3 mg	6.08	1.97	29	6.53	2.44	31	7.2%	-0.20 [-0.71, 0.31]	2009
Vorhees 2011 3 mg	22	15.99	20	25.45	15.72	20	7.0%	-0.21 [-0.84, 0.41]	2011
Warren 2011 5 mg	30.77	9.3	7	34.36	12.34	7	6.1%	-0.31 [-1.36, 0.75]	2011
Amodeo 2015 Female 5 mg	48.58	13.74	9	46.77	10.55	9	6.4%	0.14 [-0.78, 1.07]	2015
Amodeo 2015 Male 5 mg	44.42	13.1	9	43.15	8.31	9	6.4%	0.11 [-0.81, 1.04]	2015
Sadegzadeh 2020 Male 5 mg	40.61	24.96	8	38.47	17.14	8	6.3%	0.09 [-0.89, 1.08]	2020
Sadegzadeh 2020 Female 5 mg	49.17	17.89	8	29.91	17.89	8	6.1%	1.02 [-0.04, 2.08]	2020
Zolfaghari 2021 5 mg	14.25	5.19	7	36.46	6.79	7	4.4%	-3.44 [-5.27, -1.61]	2021
**Subtotal (95% CI)**			**133**			**133**	**56.7%**	**-0.70 [-1.68, 0.29]**	
Heterogeneity: Tau² = 2.00; Chi² = 93.98, df = 8 (P < 0.00001); I² = 91%									
Test for overall effect: Z = 1.39 (P = 0.16)									
**Total (95% CI)**			**202**			**202**	**100.0%**	**-0.77 [-1.36, -0.17]**	
Heterogeneity: Tau² = 1.20; Chi² = 104.13, df = 15 (P < 0.00001); I² = 86%									
Test for overall effect: Z = 2.53 (P = 0.01)									
Test for subgroup differences: Chi² = 0.03, df = 1 (P = 0.86), I² = 0%									

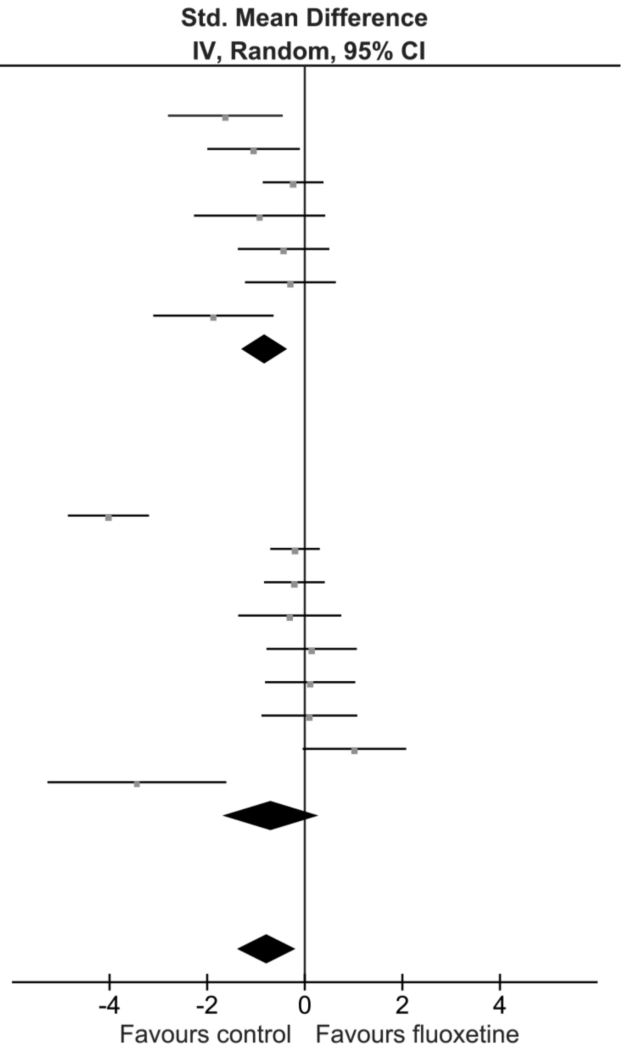

Forest plot of effects of chronic fluoxetine at higher (7.5-20 mg/kg/day) or lower (3-5 mg/kg/day) doses vs. vehicle on anxiety-like behavior in naive animals measured as time spent in the open arms of the EPM. Note: Significance of the results was unchanged when effects of fluoxetine in the dose of 20 mg instead of 10 mg from Amodeo's et al. study were included in the higher dose subgroup (Online Resource ESM_4).
