# Supplementary material for: Effects of chronic fluoxetine treatment on anxiety- and depressive-like behaviors in adolescent rodents – systematic review and meta-analysis
Source: Pharmacol Rep. 2022 Sep 24;74(5):920–46. doi: 10.1007/s43440-022-00420-w (PMC9584991; doi:10.1007/s43440-022-00420-w)
Supplement: Supplementary file 6 — Forest plot of the effects of chronic fluoxetine at higher (7.5-20 mg/kg/day) or lower (3-5 mg/kg/day) doses vs. vehicle on despair-like behavior in naïve animals measured as immobility time in the FST (PDF 1317 KB) [file 43440_2022_420_MOESM6_ESM.pdf]

Study or Subgroup	Fluoxetine Mean	Fluoxetine SD	Fluoxetine Total	Control Mean	Control SD	Control Total	Weight	Std. Mean Difference IV, Random, 95% CI	Year
**3.2.1 Higher doses**									
Iñiguez 2010 20 mg	130.01	20.68	5	178.77	27.64	5	3.5%	-1.80 [-3.41, -0.20]	2010
Vorhees 2011 10 mg	169.21	71.08	20	161.26	69.28	19	7.4%	0.11 [-0.52, 0.74]	2011
Homberg 2011 12 mg	97.66	47.38	10	65.49	24.07	10	6.0%	0.82 [-0.10, 1.74]	2011
Yoo 2013 10 mg	146.08	36.18	5	64.79	43.1	5	3.5%	1.85 [0.23, 3.46]	2013
Badenhorst 2017 10 mg	239.96	25.7	15	249.08	23.47	15	7.0%	-0.36 [-1.08, 0.36]	2016
Sonei 2017 7.5 mg	135.25	10.28	8	109.89	30.11	8	5.4%	1.07 [-0.00, 2.13]	2016
Schoeman 2017 10 mg	195.13	25.76	7	190.71	26.28	8	5.6%	0.16 [-0.86, 1.18]	2017
Yu 2022 20 mg	23.91	19.58	12	27.83	24.1	12	6.6%	-0.17 [-0.97, 0.63]	2022
**Subtotal (95% CI)**			**82**			**82**	**45.1%**	**0.19 [-0.34, 0.72]**	
Heterogeneity: Tau² = 0.33; Chi² = 17.34, df = 7 (P = 0.02); I² = 60%									
Test for overall effect: Z = 0.71 (P = 0.48)									
**3.2.2 Lower doses**									
Oh 2009 B6 strain 3 mg	78.05	10.91	36	82.33	9.41	34	8.1%	-0.41 [-0.89, 0.06]	2009
Oh 2009 SW strain 3 mg	54.72	9.93	29	44.48	7.95	31	7.8%	1.13 [0.58, 1.68]	2009
Vorhees 2011 3 mg	154.64	69.8	21	161.26	69.28	19	7.5%	-0.09 [-0.71, 0.53]	2011
Warren 2011 5 mg	101.83	25.2	7	152.38	25.2	7	4.4%	-1.88 [-3.21, -0.55]	2011
Bouet 2012 5 mg	129.57	34.95	10	125.81	37.1	10	6.3%	0.10 [-0.78, 0.98]	2012
Schoeman 2017 5 mg	148.23	25.76	7	190.71	26.28	8	4.9%	-1.54 [-2.73, -0.34]	2017
Masrour 2018 5 mg	81.66	13.96	7	88.54	22.54	7	5.4%	-0.34 [-1.40, 0.71]	2018
Sahafi 2018 5 mg	79.72	29.61	6	90.02	27.02	6	5.1%	-0.34 [-1.48, 0.81]	2018
Zolfaghari 2021 5 mg	90.05	27.19	7	99.84	19.42	7	5.4%	-0.39 [-1.45, 0.67]	2021
**Subtotal (95% CI)**			**130**			**129**	**54.9%**	**-0.31 [-0.88, 0.26]**	
Heterogeneity: Tau² = 0.53; Chi² = 34.18, df = 8 (P < 0.0001); I² = 77%									
Test for overall effect: Z = 1.08 (P = 0.28)									
**Total (95% CI)**			**212**			**211**	**100.0%**	**-0.07 [-0.46, 0.31]**	
Heterogeneity: Tau² = 0.42; Chi² = 52.64, df = 16 (P < 0.00001); I² = 70%									
Test for overall effect: Z = 0.37 (P = 0.71)									
Test for subgroup differences: Chi² = 1.61, df = 1 (P = 0.20), I² = 38.0%									

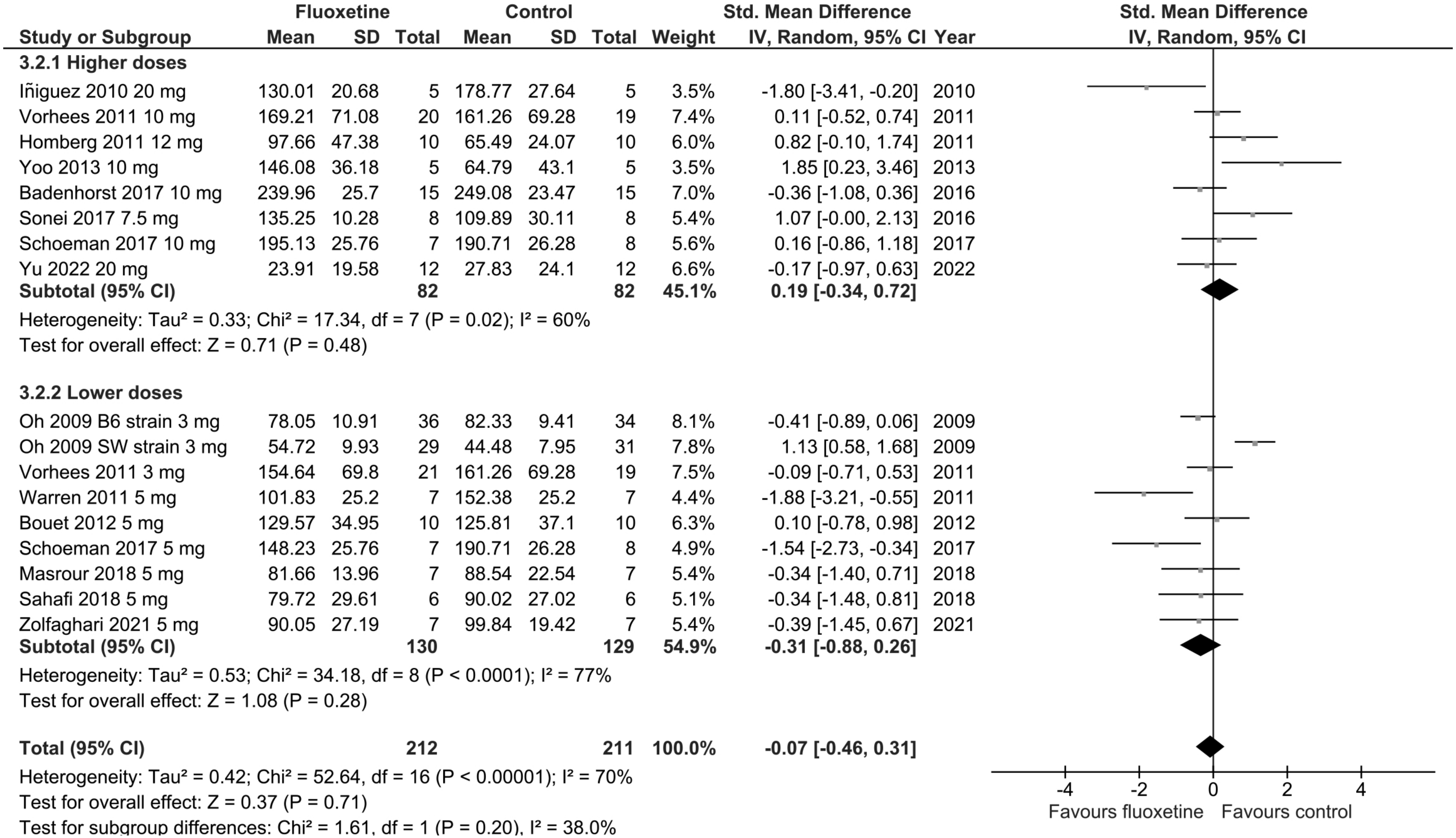

Forest plot of the effects of chronic fluoxetine at higher (7.5-20 mg/kg/day) or lower (3-5 mg/kg/day) doses vs. vehicle on depressive-like behavior in naive animals measured as immobility time in the FST.
